# Edible Matrix Code with Photogenic Silk Proteins

**DOI:** 10.1021/acscentsci.1c01233

**Published:** 2022-04-13

**Authors:** Jung Woo Leem, Hee-Jae Jeon, Yuhyun Ji, Sang Mok Park, Yunsang Kwak, Jongwoo Park, Kee-Young Kim, Seong-Wan Kim, Young L. Kim

**Affiliations:** † Weldon School of Biomedical Engineering, Purdue University, West Lafayette, Indiana 47907, United States; ‡ Department of Mechanical System Engineering, Kumoh National Institute of Technology, 61 Daehak-ro, Gumi-si, Gyeongsangbuk-do 39177, Republic of Korea; § Department of Agricultural Biology, National Institute of Agricultural Sciences, Rural Development Administration, Wanju, Jeollabuk-do 55365, Republic of Korea; ∥ Purdue University Center for Cancer Research, West Lafayette, Indiana 47907, United States; ⊥ Regenstrief Center for Healthcare Engineering, West Lafayette, Indiana 47907, United States; # Purdue Quantum Science and Engineering Institute, West Lafayette, Indiana 47907, United States

## Abstract

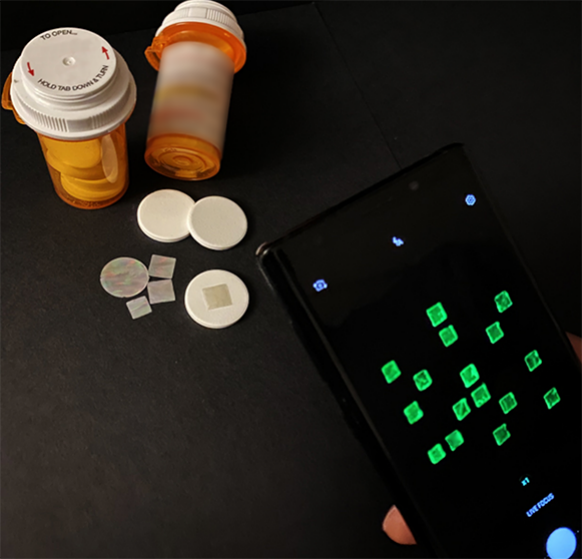

Counterfeit medicines are a healthcare security problem, posing not only a direct threat to patient safety and public health but also causing heavy economic losses. Current anticounterfeiting methods are limited due to the toxicity of the constituent materials and the focus of secondary packaging level protections. We introduce an edible, imperceptible, and scalable matrix code of information representation and data storage for pharmaceutical products. This matrix code is digestible as it is composed of silk fibroin genetically encoded with fluorescent proteins produced by ecofriendly, sustainable silkworm farming. Three distinct fluorescence emission colors are incorporated into a multidimensional parameter space with a variable encoding capacity in a format of matrix arrays. This code is smartphone-readable to extract a digitized security key augmented by a deep neural network for overcoming fabrication imperfections and a cryptographic hash function for enhanced security. The biocompatibility, photostability, thermal stability, long-term reliability, and low bit error ratio of the code support the immediate feasibility for dosage-level anticounterfeit measures and authentication features. The edible code affixed to each medicine can serve as serialization, track and trace, and authentication at the dosage level, empowering every patient to play a role in combating illicit pharmaceuticals.

## Abstract 2

An edible code affixed to each medicine offers anticounterfeit measures and authentication features at the dosage level, empowering every patient to play a role in combating illicit pharmaceuticals.

## Introduction

Pharmaceutical products are the most vulnerable goods for widespread illicit activities. The concept of counterfeit medicines includes substandard, falsified, and diverted ones in a broad manner.^1,2^ This problem is not new but is increasingly becoming a tremendous burden to society regardless of a country’s economic status. Counterfeited medication not only poses a serious threat to patient safety and public health but also causes heavy economic losses, accounting for 10% of the global pharmaceutical trade and $200 billion annually.^3−8^ For example, counterfeit malaria and pneumonia medicines are responsible for 250 000 child deaths per year.^9^ One hundred fifty people in Singapore were hospitalized after the administration of counterfeit erectile dysfunction medicines.^10^ In Hong Kong, 40% of Viagra sales are counterfeits.^11^ The opioid crisis in the U.S. has triggered counterfeit opioid production, which has caused deaths in almost all states.^12,13^ Africa is the region most affected; 42% of all counterfeit medicines reported between 2013 and 2017 have African origins.^4,14,15^ Counterfeit medicines of both lifestyle and lifesaving drugs are progressively common in the U.S.^5,8,16−18^ In addition, counterfeiting of pharmaceutical products can constitute an infringement of intellectual property rights, undermining brand names, scientific innovations, and financial rewards.

Counterfeit medicines are attributable to the increased use of online (or Internet) pharmacies.^18,19^ Thirty-five thousand to 40 000 active online pharmacies worldwide, with 600 added every month, sell medicines to patients.^20,21^ Surprisingly, it is estimated that 96–97% of these online pharmacies operate illegally.^22,23^ Illegal online pharmacies are a public health issue affecting the quality and authenticity of medications supplied. A surge in counterfeit treatments and medical supplies has recently occurred during the ongoing pandemic (e.g., counterfeit chloroquine and hydroxychloroquine).^24−26^ Indeed, the illicit activities related to COVID-19 have been further worsened during the pandemic. In addition, more than 12% of illegal online pharmacies are found to sell controlled substances without a prescription, contributing to the opioid epidemic even among high school and college students.^27^ Unfortunately, even though public resources are available for consumers and end users,^28,29^ most patients and healthcare providers (physician, nurse, and pharmacist) are not well informed to avoid the unintentional use of illegal online pharmacies.

Various measures at the packaging level have been used to ensure the integrity of pharmaceutical products and to combat counterfeiting to some extent. Major pharmaceutical companies commonly use anticounterfeit methods that focus on secondary packaging (i.e., exterior box) level protections, which group a certain number of products and employ track-and-trace measures outside primary packaging. Typically, they utilize barcodes, quick response (QR) codes, holograms, and radiofrequency identification for brand protection and to preserve product integrity.^30−34^ In the U.S., the Drug Supply Chain Security Act requires unit-level traceability by 2023.^35^ In Europe, the Falsified Medicines Directive requires the implementation of safety features.^2^ As a result, major pharmaceutical manufacturers, distributors, and retailers in the U.S. have recently created a blockchain-based solution for supply chain management (also known as the MediLedger Network).^36^

Advanced anticounterfeit technologies should focus on individual medicines at the dosage level. In retail and hospital pharmacy settings, medicines are separated from the original packaging and are placed into individual doses for patient safety, quality control, delivery, and inventory tracking. The existing dosage-level protections include QR code drug labels,^37,38^ silica microtaggants,^39^ DNA taggants,^39,40^ polymer molecular encoding,^41^ isotope-labeled excipients,^42^ multicolor nonpareil coatings,^43^ watermark bioprinting,^44^ and metal nanoparticle taggants.^45−47^ Fluorescent material-based taggants are also an attractive alternative,^48−57^ because fluorescence signals can be read under external stimuli while controlling certain encoding parameters. As a result, several fluorescent materials have been applied for anticounterfeiting medicine technologies,^58^ including barcoded microfibers using coumarin-6,^59^ rhodamine B microtaggants on polyethylene glycol,^60^ QR-coded capsules using upconversion fluorescent nanoparticles,^61^ dextran-modified 2-hydroxyethyl methacrylate polymer particles,^62^ and lysozyme supramolecular nanofilms.^63^

However, the currently available anticounterfeit measures and authentication features have several limitations. First, the existing security measures implemented at the supply chain level (or secondary packaging) are not intended to provide patients with the ability to verify their own medicines. Second, the on-dose technologies require skilled and trained personnel as well as high-cost sophisticated analyzers and specialized readers. Third, simple on-dose taggants or labels (e.g., barcode, QR code, and multicolor coating) can easily be replicated or tampered. Simply, the secondary packaging (i.e., exterior box) is highly vulnerable. Finally, the commonly used materials are not ideal for oral intake because foreign and artificial additives can potentially have hazardous and adverse (e.g., carcinogenic and cytotoxic) consequences.^64,65^ There is also emerging concern about pharmaceutical coating materials (e.g., phthalate) as endocrine disruptors.^66^

On-dose authentication for solid oral-dosage forms or in-dose authentication for liquid dosage forms at the point of medication administration can offer the highest protection, overcoming the limitations of current anticounterfeiting and authentication methods for pharmaceuticals. On-dose authentication means that a security measure is directly integrated with the dosage form itself, offering product verification and traceability embedded into each medicine.^39,67−69^ Even if separated from the secondary package, every individual medicine can be verified and authenticated independently. On-dose authentication allows patients to verify their medicines in real time with important dose information. Pharmaceutical companies or hospital pharmacies can implement serialization directly on individual medicines for brand protection while providing for secure administration at the patient bedside. Researchers who are conducting clinical trials can ensure that participants are properly self-administering clinical trial medication at home.

In this paper, we introduce all protein-based matrix codes for pharmaceutical anticounterfeiting and on-dose (or in-dose) authentication. First, we utilize silk fibroin genetically hybridized with multiple distinct fluorescent proteins as the constituent materials for the proposed matrix codes. Second, we develop a simple fabrication method for generating imperceptible multidimensional codes with a variable capacity to encode information in a manner similar to conventional barcodes or QR codes. Third, after establishing a cryptographic key extraction protocol with a deep learning method, we use a smartphone to demonstrate on-dose and in-dose authentication of an oral-dosage medicine and an alcohol dosage form under simulated settings, respectively. Fourth, we study the digestibility of the proposed edible codes using proteolytic enzymes released in the gastrointestinal tract for protein denaturation and degradation. Finally, we also characterize the biocompatibility, photostability, thermal stability, and long-term reliability of the edible codes. Overall, the proposed all protein-based matrix codes applying to individual medicines can provide patients with the last line of defense, empowering them to play a key role in combating counterfeit medicines and avoiding the unintentional use of counterfeit medicines.

## Results and Discussion

**Figure 1:**
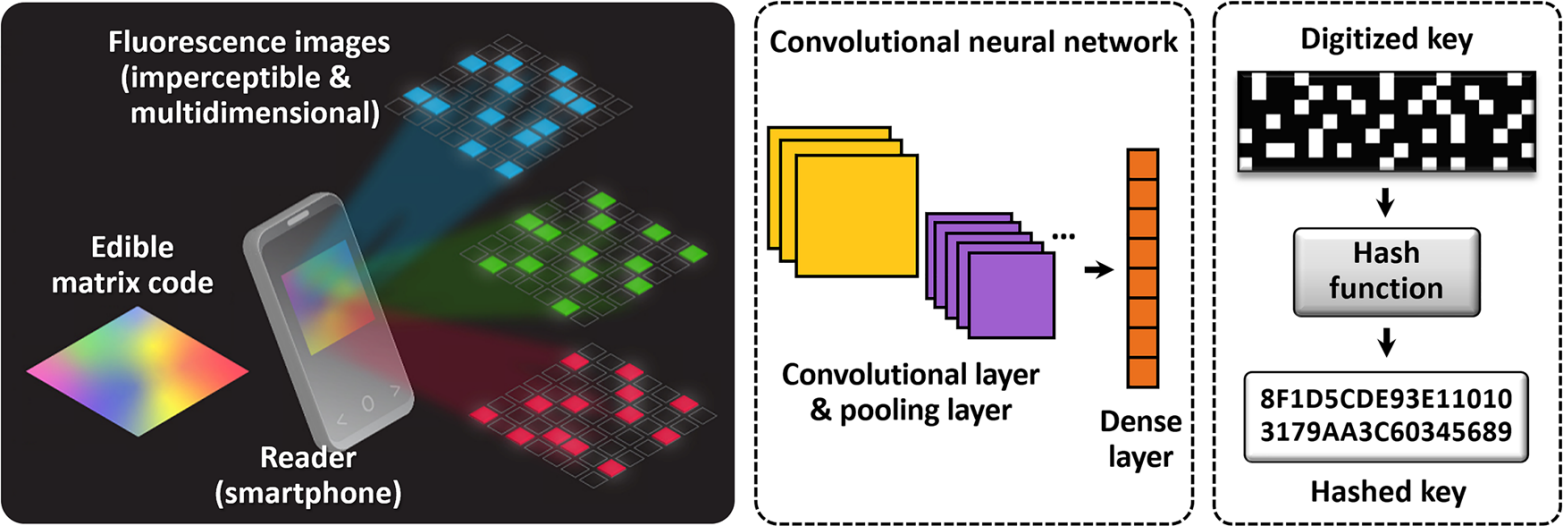
Schematic illustration of an edible matrix code for anticounterfeiting pharmaceutical products and on-dose (or in-dose) authentication. Like other machine-readable barcodes or 2D matrix codes (e.g., QR code), the proposed code is a method of digital information representation and data storage but has unique features to be edible, imperceptible, and multidimensional, using three different fluorescent natural biopolymers (i.e., silk fibroin and fluorescent protein). After being affixed to an individual medicine by the pharmaceutical manufacturer or the hospital pharmacy, this code becomes an integrated part of the medicine. The end user or consumer (e.g., patient) can use a smartphone camera to read the code (i.e., fluorescence images). A digitized key is generated from the code by a deep neural network for quick and accurate key extraction. The extracted digital key is converted to a cryptographic hashed key through a hash function. As a result, the patient can be empowered to authenticate the medicine with the necessary dose information immediately before oral intake.

Figure 1 illustrates the proposed code representing a digital key for anticounterfeiting medicines with on-dose (or in-dose) authentication. This code is similar to other machine-readable barcodes and two-dimensional (2D) matrix codes (e.g., QR code) but is unique in that it is edible, imperceptible, and multidimensional. From a materials standpoint, this code is composed of all proteins (i.e., silk fibroin and fluorescent protein) without using synthetic polymers or materials. This code becomes an integrated part of the medicine after being affixed to an individual solid oral-dosage form (e.g., pill, tablet, or capsule). From a security standpoint, multiple distinct fluorescence emission colors can be incorporated into a multidimensional parameter space to enhance an encoding capacity as well as attack resistance. This code is imperceptible because the fluorescence emission is detected only by a unique set of excitation and emission optical filters. To overcome pattern and shape imperfections occurring during the fabrication process, a deep neural network is applied to extract a digitized key. The digitized key is then converted to a cryptographic hashed key through a hash function. From a patient standpoint, the patient (or end user or consumer) can use a smartphone camera as a reader to scan the code and to authenticate the medicine with the extracted security key immediately before oral intake.

Safe on-dose authentication would be possible only if the materials encoded with digital information are edible, digestible, and free of any toxic or cytotoxic elements. Silk protein (i.e., fibroin) produced by silkworms (*Bombyx mori*) is an excellent choice for a natural biopolymer. Silk fibroin is biocompatible with low immunogenicity, resulting in minimal inflammatory and immune responses.^70−72^ Silk fibroin is composed of amino acids including glycine, alanine, serine, lysine, arginine, and leucine.^73−75^ The protocols for extracting silk fibroin without introducing heavy metals and toxic trace elements are well developed.^69,76−78^ Silk proteins are currently approved for a wide range of food applications and are generally recognized as safe (also known as GRAS) as designated by the U.S. Food and Drug Administration. Simply, it is edible and digestible.^69,79,80^

Recombinant silk proteins can be mass-produced with several host systems.^81^ Specifically, genetic fusion of silk fibroin and fluorescent protein is readily available via *piggyBac* transposase and clustered regularly interspaced short palindromic repeats (CRISPR) tools.^82−85^ These biomanufacturing methods are highly ecofriendly, scalable, and sustainable.^81,86,87^ Fluorescent proteins are often included in genetically modified dietary products for oral consumption.^88^ When compared with common food allergens, fluorescent proteins do not have common allergen epitopes and are well degraded during gastric digestion.^89^ Fluorescent silk fibroin can easily be processed into polymeric materials for fabricating a variety of types of rigid or flexible structures with tunable mechanical and optical properties.^69,90,91^ Modernized silkworm farming (i.e., sericulture) can potentially offer a sustainable, scalable, and ecofriendly production strategy of such recombinant proteins in an economical and industrially relevant manner without consuming fossil fuels and raw materials.^81,86^

**Figure 2:**
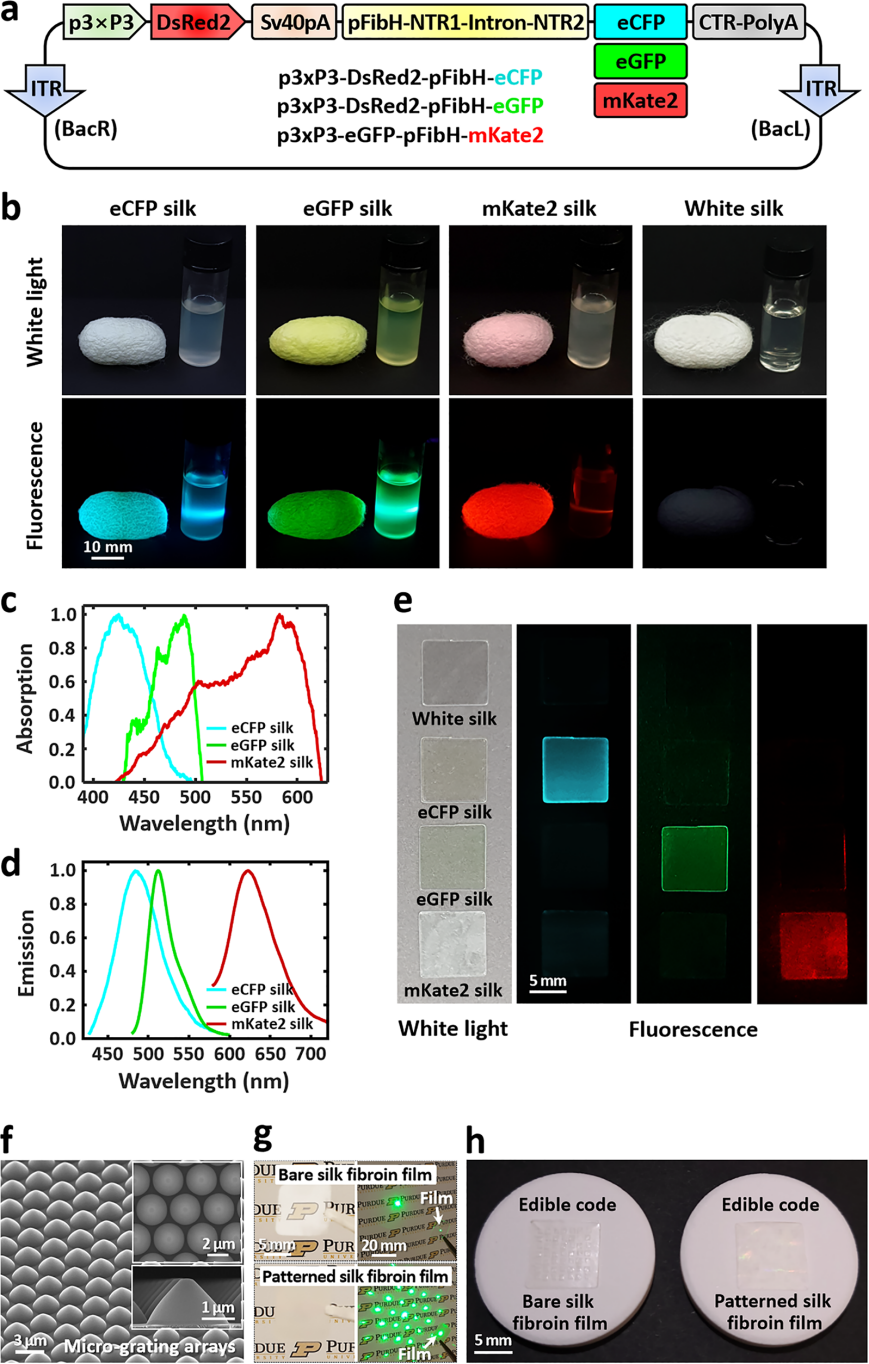
Fabrication of edible matrix codes using silk fibroin genetically hybridized with fluorescent proteins. (a) Schematic representation of transformation vector structures for silkworm transgenesis, p3×P3-DsRed2-pFibH-eCFP for eCFP silk, p3×P3-DsRed2-pFibH-eGFP for eGFP silk, and p3×P3-eGFP-pFibH-mKate2 for mKate2 silk. Fibroin heavy chain promoter domain (pFibH, 1124 base pairs (bp)), N-terminal region 1 (NTR1, 142 bp), Intron (871 bp), N-terminal region 2 (NTR2, 417 bp), C-terminal region (CTR, 179 bp), poly(A) signal region (PolyA, 301 bp), enhanced cyan fluorescent protein (eCFP, 720 bp), enhanced green fluorescent protein (eGFP, 720 bp), monomeric far-red fluorescent protein (mKate2, 699 bp), inverted repeat sequences of *piggyBac* arms (ITR), 3×P3 promoter (273 bp), and Sv40 polyadenylation signal sequence (Sv40pA, 268 bp). Red fluorescent protein (DsRed2) is used only for a marker gene of eCFP and eGFP, while eGFP is utilized for a marker gene of mKate2. (b) Photographs and fluorescence images of eCFP silk, eGFP silk, and mKate2 silk cocoons, compared with a nontransgenic (wild-type) white silk cocoon. Each silk fibroin solution is regenerated from the corresponding silk cocoons. (c, d) Optical absorption (c) and fluorescence emission (d) spectra of fluorescent silk fibroin films fabricated using the regenerated eCFP silk (cyan), eGFP silk (green), and mKate2 silk (red) fibroin solutions. (e) Photographs and fluorescence images of three different fluorescent silk fibroin films and a white silk fibroin film using an appropriate set of optical excitation and emission. A set of an excitation source (λ_ex_) and an emission filter (λ_em_) is used as follows: λ_ex_ = 415 nm and λ_em_ = 460 nm, λ_ex_ = 470 nm and λ_em_ = 525 nm, and λ_ex_ = 530 nm and λ_em_ = 630 nm for eCFP silk, eGFP silk, and mKate2 silk, respectively. The thickness of the fluorescent silk fibroin films is 70 μm on average. (f) Scanning electron microscopy images of conical micrograting arrays with 2D periodic hexagonal patterns. The height and bottom diameter of each grating are 1.4 and 2.7 μm with a distance (i.e., period) between adjacent gratings of 2.9 μm. (g) Photographs of light propagation (green laser at λ = 532 nm) through bare (top) and micrograting patterned (bottom) silk fibroin films. (h) Photograph of 7 × 7 matrix codes fabricated using bare (left) and micrograting patterned (right) silk fibroin films, affixed onto the tablet-type medicine (oral solid dosage). The code pattern on the micrograting patterned silk fibroin film is covert and imperceptible due to the strong diffraction of light caused by the micrograting arrays.

We take advantage of three different fluorescent silk recombinants of enhanced cyan fluorescent protein (eCFP), enhanced green fluorescent protein (eGFP), and far-red fluorescent protein (mKate2). The genetic hybridization of silk with fluorescent proteins is conducted by the *piggyBac* transposase method (see Methods). To produce eCFP silk, eGFP silk, and mKate2 silk, each fluorescent protein gene is fused with N-terminal and C-terminal domains of the fibroin heavy (H)-chain promoter (pFibH), creating p3×P3-DsRed2-pFibH-eCFP, p3×P3-DsRed2-pFibH-eGFP, and p3×P3-eGFP-pFibH-mKate2 transformation vectors, respectively (Figure 2a). Each transition vector is injected with a helper vector into preblastoderm embryos of silkworms to produce transgenic fluorescent silkworms that spin eCFP silk, eGFP silk, and mKate2 silk cocoon fibers (Figure 2b and Figure S1). The fluorescent silk cocoons are further processed into polymeric solutions by minimizing the heat-induced denaturation of fluorescent proteins in silk (see Methods and Supporting Information). The eCFP silk, eGFP silk, and mKate2 silk fibroin solutions and films have cyanic, green, and red fluorescence emission colors, respectively, each of which requires a unique set of optical excitation and emission wavelength bands (λ_ex_ and λ_em_) in the visible region (Figure 2b–e).

To develop a three-dimensional (3D) matrix code of distinct fluorescence emission colors, four-matrix code patterns with predetermined openings are formed on a thin nonfluorescent white silk fibroin film (Figure S2). The four opening patterns are not overlapped, resulting in one of four levels (three fluorescence colors and none) in each square code unit (size ≈ 700 × 700 μm^2^) (Figure S3a,b). Importantly, this multidimensionality can enhance the parameter space to be tamper-resistant and to implement postprocessing for specific applications.^69,92,93^ The fluorescent matrix code pattern formed on a bare white silk fibroin film can be visible, depending on a view angle, although the type of silk fibroin (i.e., white, eCFP, eGFP, or mKate2) is not distinguishable to the naked eye (Figure S3c). To further enhance the invisibility of the embedded code array, the white silk fibroin film is patterned with conical micrograting arrays via soft imprint lithography (Figure 2f and Figure S2a). The geometry (shape and arrangement) of micrograting arrays is designed to generate strong optical diffraction (i.e., light scattering) to mask the embedded code array pattern (Figure 2g,h and Figure S4).^94,95^ Using the reported fabrication method, 5 × 5, 7 × 7, and 9 × 9 matrix arrays can be embedded in a taggant size of 7 × 7 mm^2^, 9 × 9 mm^2^, and 11 × 11 mm^2^, respectively. Such a different number of matrix arrays results in a variable encoding capacity (Figure S5). As a number of possible output keys generated by an input matrix array, the encoding capacity of edible matrix codes can be defined as *c*^*s*^ where *c* is the bit-level (*c* = 2 for binary bits) of keys, and *s* is the key size.^69,93^

We also explore if the proposed edible code can be used for liquid dosage forms because the current anticounterfeit technologies are limited for liquid medicines.^58^ Inspired by the idea that silk fibroin extensively treated with alcohol has enhanced optical and mechanical properties, we focus on alcohol-containing medicines.^96−98^ Surprisingly, alcohol (i.e., ethanol) is often a common ingredient in liquid dosage form medicines (e.g., syrups, solutions, and emulsions). Indeed, some liquid medicines contain a high level of alcohol.^99−101^ In this respect, we test the morphological and photoluminescence properties of fluorescent silk fibroin films in 200 proof ethanol solutions at various concentrations and exposure periods (Figure S6). Importantly, the ability of eGFP silk fibroin films for emitting the fluorescence is not strongly affected by ethanol at any of these concentrations, although swelling occurs in water and 10% ethanol solutions due to the formation of new hydrogen bonds.^102^ On the other hand, the fluorescent silk films do not undergo significant deformation at alcohol concentrations higher than 20% (v v^–1^) even over 10 months. This is attributable to the increased crystallinity as random coils (i.e., silk I) are converted into β-sheets (i.e., silk II).^96,103,104^ In other words, the proposed edible code can be applied to liquid medicines containing a high alcohol content (>20%) for in-dose authentication.

**Figure 3:**
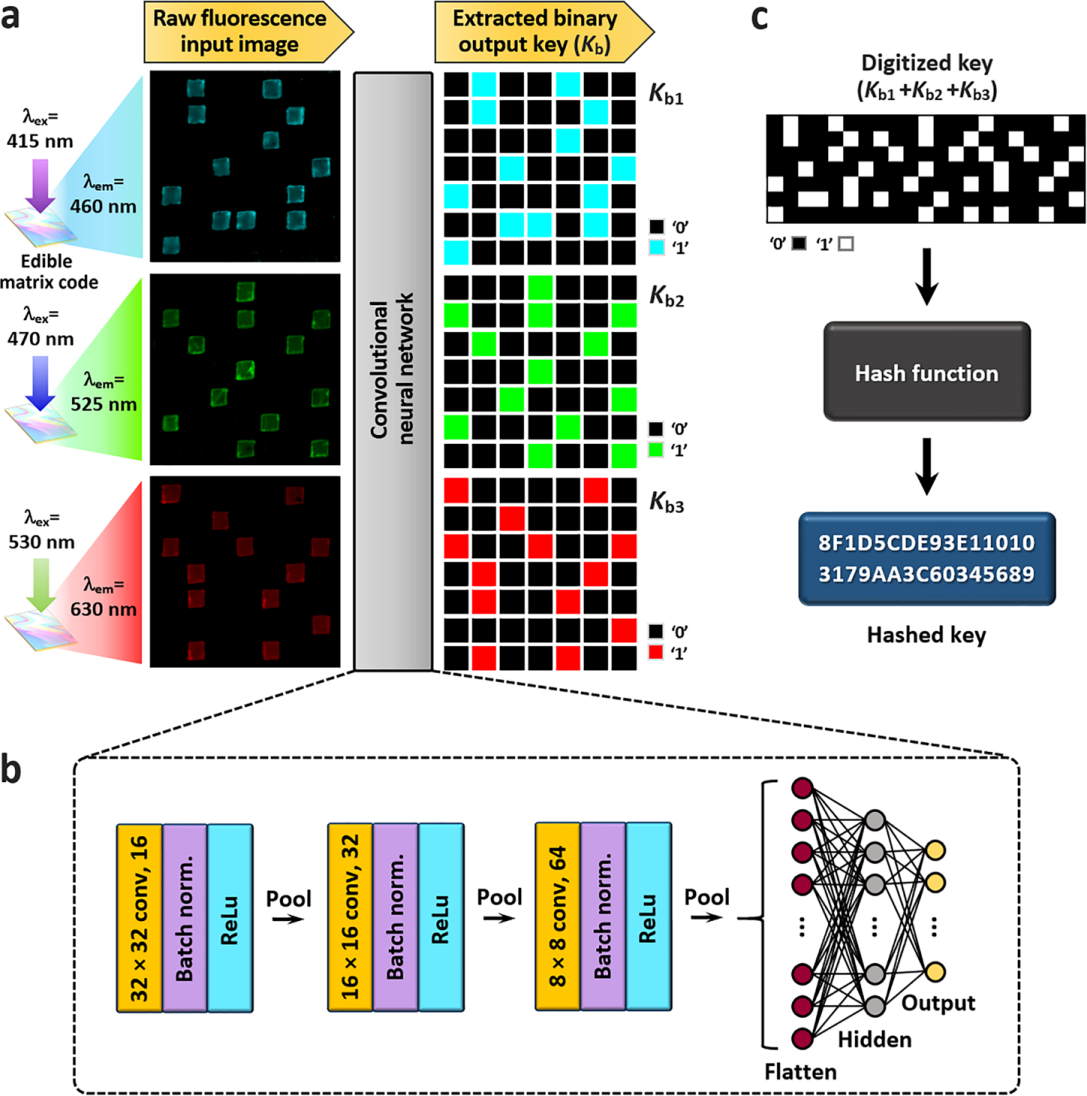
Cryptographic key generation of an edible code with three distinct fluorescence colors and digital signature generation with a hash algorithm. (a) Extraction process of digitized output keys from raw fluorescence input images of a representative edible code (7 × 7 matrix). Three different fluorescence images are acquired with an optical set of excitation and emission: eCFP silk code pattern (cyan); λ_ex_ = 415 nm and λ_em_ = 460 nm, eGFP silk code pattern (green); 470 and 525 nm, and mKate2 silk code pattern (red); 530 and 630 nm. Each code pattern generates a 49-bit long binary key *K*_b_. The binary keys of three different codes are combined to a digitized key of 147 bits (*K*_b1_ + *K*_b2_ + *K*_b3_). In the case of a 7 × 7 matrix code, the nominal encoding capacity is calculated to be 2^147^ (≈ 1.78 × 10^44^). (b) Convolutional neural network (CNN) architecture for output key extraction of an edible matrix code. A 2D CNN model consists of three convolutional layers and two fully connected layers (Table S1). Batch normalization is applied to each convolutional layer for faster and more stable training. After each batch normalization, the rectified linear unit (ReLU) activation function is applied, and max-pooling is performed. (c) Hashed key generation from the extracted digitized key via a cryptographic hash algorithm (e.g., MD5). Other strong hash functions can be used including SHA-256 and SHA-512. A hashed key can be used for authentication, ensuring key integrity and securing against unauthorized modifications.

To reliably extract a digitized key from an edible matrix code, we use a 2D convolutional neural network (CNN) that takes raw fluorescence images of the code as an input and returns a binary output key of each fluorescence emission color (Figure 3a). Compared with classical imaging processing, this deep learning method is beneficial to overcome imperfections of resultant code patterns undesirably formed during the fabrication. First, raw fluorescence images of an edible code are acquired by a camera through an optical set of an excitation source and an emission filter (eCFP silk: λ_ex_ = 415 nm and λ_em_ = 460 nm; eGFP silk: 470 and 525 nm; and mKate2 silk: 530 and 630 nm). Second, the 2D CNN is designed to have a series of convolutional and pooling layers and fully connected layers at the end (Methods, Figure 3b, and Table S1). This model is trained to detect filled square units as 1’s and empty areas as 0’s in a matrix array, extracting a binary output key (*K*_b_) from each fluorescence emission color image. To train the model, 200 individual square units (each square unit code size = 101 pixels ×101 pixels) are acquired from the fluorescence images of the fabricated edible matrix codes (Figure S7). The square code units are randomly selected to generate 9494 different synthetic code images in a format of 7 × 7 matrix array (each image size = 692 pixels × 648 pixels), which serve as a training data set. To quantitatively validate the designed 2D CNN model, 50 000 synthetic input images (7 × 7 matrix) are fed into the model to extract binary output keys, resulting in a low bit error ratio of 1.62 × 10^–4^ (Figure S8). In the case of a 7 × 7 matrix code, the resultant digitized key size (*K*_b1_ + *K*_b2_ + *K*_b3_) is 147 (= 49 × 3) bits. For 5 × 5 and 9 × 9 matrix codes, the digitized key sizes are 75 and 243 bits, respectively (Figure S9). Third, to aid secure authentication and ensure data integrity, we employ a hash function that returns a unique digital signature (i.e., hashed key) from the extracted digitized key in an irreversible (one-way) manner (Figure 3c).^105^ As an example, the final extracted key is entered into the MD5 message-digest algorithm, producing a hashed key with 128 bits.^106^ Ultimately, this hashed key can be used for validation, verification, and authentication for individual medicines. The extracted binary key (*K*_b_) can also be reconstructed to a quaternary key and a double binary key (Figure S10).

**Figure 4:**
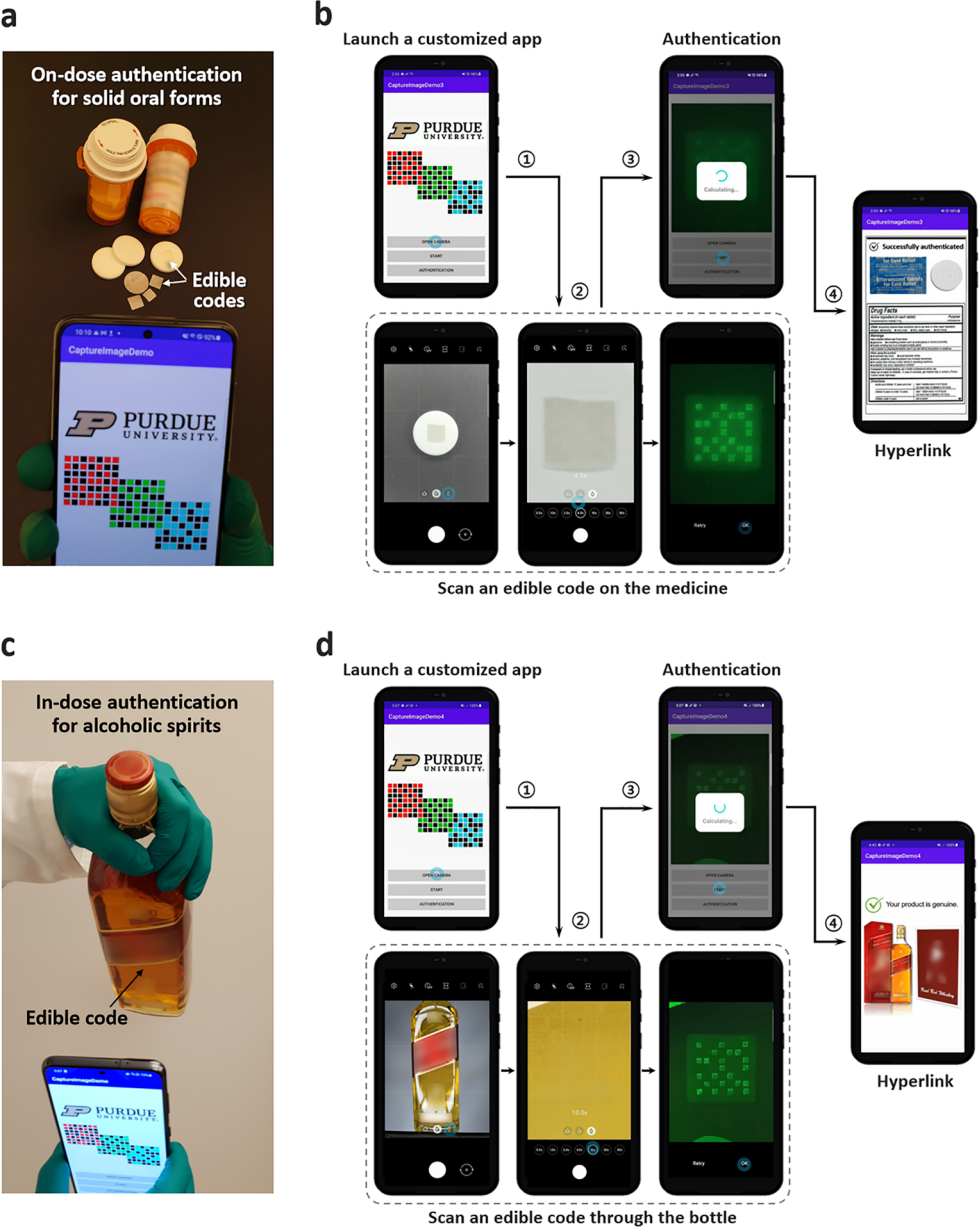
Edible code applications for authentication using a smartphone. (a, b) Simulated on-dose authentication of medicines. (a) Photograph of on-dose authentication of medicines integrated with an edible code. (b) Simulated authentication process for an oral-dosage tablet-type medicine. A custom-built mobile application (app) consists of the following steps for an end user or consumer (Movie S1): launch the customized app, scan an edible code using a set of excitation (470 nm) and emission (525 nm) optical filters (Figure S11). Then, this mobile app authenticates the scanned code and further opens the embedded hyperlink to a webpage to confirm the genuine medicine information, such as product data (e.g., dosage strength, dose frequency, cautions, and expiration date), manufacturing details (e.g., location, date, batch, and lot number), and distribution path (e.g., country, distributor, and wholesaler). (c, d) Bottle-through edible code application for in-dose authentication of high-value alcoholic spirits. (c) Photograph of simulated in-dose authentication of a Scotch whisky bottle (e.g., 80 proof whisky, 40% alcohol per volume) that contains an edible code inside. To image the edible code through the bottle, the bottle is titled facing down. (d) Simulated authentication process for an alcoholic spirit containing an edible code inside. The customized mobile app can authenticate the scanned code and further inform the genuine product information (Movie S2), such as product data (e.g., type, ingredients, alcohol concentration, and cautions), manufacturing details (e.g., location, date, and serial number), and distribution path (e.g., country, distributor, and wholesaler).

We demonstrate a smartphone-based on-dose authentication application of an edible code affixed to an oral-dosage tablet-type medicine in a simulated dose setting (Figure 4a). For simplicity and practicality, a custom-built mobile application (app) is designed to scan an edible code generating the eGFP fluorescence emission color with only one set of optical filters (Figure S11 and Movie S1). When the patient views the edible code on the solid medicine through the smartphone screen, the mobile app automatically recognizes the spatial pattern of the code and extracts a digitized key. After the corresponding hashed key is produced, the app opens an embedded hyperlink to the webpage for confirming authentication and showing the dose information. In addition, we test if an edible code can be imaged through the bottle (bottle-through code application) for an authentication identifier of high-value alcoholic spirits (Figure 4b and Figure S12). This simulated in-dose authentication uses a Scotch whisky bottle (e.g., 80 proof whisky, 40% alcohol per volume) in which an edible code is submerged inside (Movie S2). Using the mobile app, the consumer scans an edible code through the glass bottle without opening the bottle. After the same acquisition processing steps, the mobile app opens an embedded hyperlink to the webpage to confirm the genuine product information. The alcohol tolerance of the proposed edible codes can also be useful for fighting against high-value counterfeit alcoholic spirits, which are one of the most troublesome counterfeit products.^107−109^ For example, it is estimated that the U.K. spirits market including whiskies and gins is £5.5 billion and that 18% U.K. adults experienced purchasing counterfeit alcoholic spirits.^110,111^

**Figure 5:**
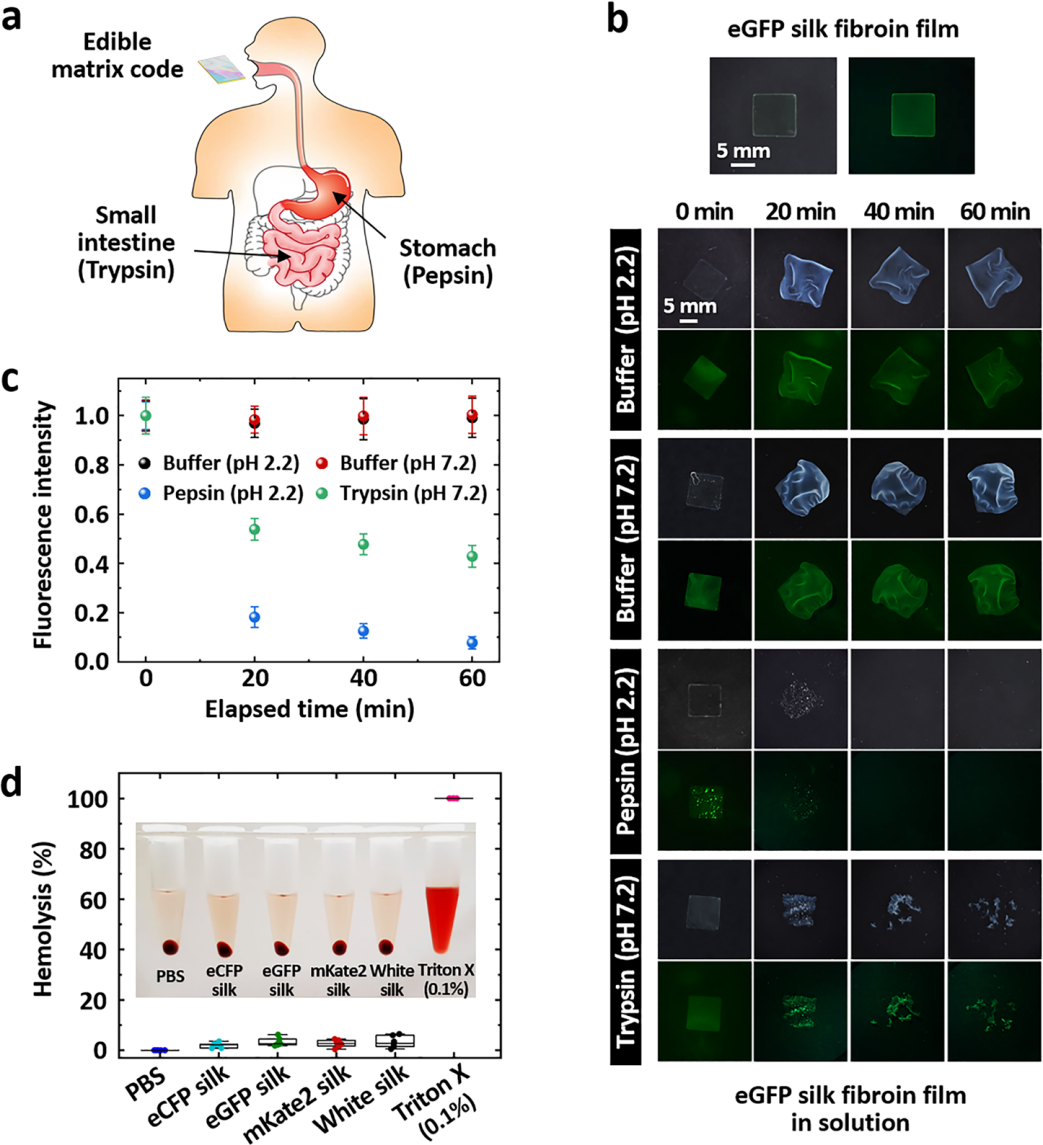
Enzymatic digestibility and biocompatibility of all protein-based matrix codes. (a) Schematic illustration of the gastrointestinal tract (the stomach and the small intestine) where pepsin and trypsin are the major proteolytic enzymes produced for denaturation and degradation of dietary proteins. (b) Photographs and fluorescence images of eGFP silk fibroin films immersed in pepsin (pH 2.2) enzyme or trypsin (pH 7.2) enzyme solutions as a function of elapsed time. For comparison, buffer solutions with the same pH values without enzymes are tested. (c) Fluorescence emission intensity of eGFP silk fibroin films immersed in the proteolytic enzyme and buffer solutions at λ = 525 nm. The fluorescence intensity is normalized by the value at 0 min. The rapid decrease in the eGFP fluorescence intensity supports the denaturation and degradation of the protein-based edible codes. The enzymatic tests were repeated four times, and the error bar is a standard deviation. (d) Red blood cell hemolysis test of different silk fibroin solutions. For comparison, 0.1% Triton X-100 and phosphate-buffered saline (PBS) without silk solutions were used as positive (hemolysis efficiency of 100%) and negative (0%) controls, respectively. Inset: representative photograph of samples using sheep erythrocytes.

To evaluate the digestibility of edible codes, we investigate enzymatic degradation rates of fluorescent silk fibroin *in vitro* using two major proteolytic enzymes produced in the gastrointestinal tract under physiologically relevant conditions (Figure 5a). Dietary protein digestion involves denaturation (i.e., protein unfolding) and degradation (i.e., primary structure destruction).^112^ Pepsin is produced in the stomach to denature food proteins as a nonspecific protease under a highly acidic environment.^113^ Trypsin produced in the pancreas is released into the small intestine to further degrade proteins at a neutral pH level.^114^ For quantifying protein denaturation and degradation, eGFP fluorescence is a reliable marker because the exact protein sequence is required to form the chromophore and eGFP fluorescence is favorably sensitive to even subtle denaturation.^115−118^ In Figure 5b, eGFP silk fibroin films (size = 9 × 9 mm^2^) are immersed in 0.1% pepsin (pH 2.2) and 0.25% trypsin (pH 7.2) solutions. A decrease in the eGFP fluorescence intensity (λ_em_ = 525 nm) averaged over an area of 15 × 15 mm^2^ is used to monitor protein denaturation and degradation of edible codes (Figure 5c). The eGFP silk fibroin films in the control solutions (pH 2.2 and pH 7.2 buffers) without enzymes maintain the strong fluorescence emission over a period of 60 min, although cloudy swelling and shape distortion occur. On the other hand, gastric enzyme exposure for the same period significantly disfigures the eGFP silk fibroin films. Expectedly, the reduction of the eGFP fluorescence intensity in pepsin is about 1.6 times faster than that in trypsin on average after 60 min (*p*-value of repeated measures ANOVA test ≈ 0).

To further examine the biological compatibility for the constituent materials of an edible code, we perform a standard hemolysis test^119^ of white silk and fluorescent silk fibroin solutions with a concentration of 5–6% (w v^–1^) using sheep erythrocytes (Figure 5d). For comparison, 0.1% Triton X-100 and phosphate-buffered saline (PBS) without silk solutions are used as positive and negative controls, respectively. The positive control (0.1% Triton X-100) clearly shows a uniform breakdown of red blood cells, whereas the silk samples and negative control (PBS) reveal a pale yellow color (inset of Figure 5d). The hemolysis efficiency is defined as (*A*_S_ – *A*_N_)/(*A*_P_ – *A*_N_) × 100 (%), where *A*_S_, *A*_P_, and *A*_N_ are the optical absorption values at λ = 580 nm for the silk samples, the positive control, and the negative control, respectively. The hemolysis efficiency values of the four silk samples are not statistically different from that of the negative control (*p*-value of ANOVA test = 0.16).

Finally, we explore the photostability, thermal stability, and long-term reliability of edible codes. The fluorescent silk fibroin films have a relatively good photostability for 210 h upon white light illumination with a high intensity of 5000 lx, which is 10 times higher than the recommended office workspace light intensity of 500 lx (Figure S13).^120^ If a currently available pharmaceutical (dark or opaque) packaging with light protection is used, the shelf life will be significantly extended. When bit error ratios of output keys extracted from edible codes are examined under heat treatments, the bit errors are negligible if the fluorescence intensity is maintained at above 75% which corresponds to 65, 65, and 60 °C for eCFP silk, eGFP silk, and mKate2 silk fibroin films, respectively (Figure S14). When an output key is re-extracted after 360 days stored at 23 ± 2 °C and 30–40% relative humidity in the dark, the bit error ratio is zero (Figure S15). Overall, the digestibility, biocompatibility, and physical stability support the idea that all protein-based edible codes can be easily and safely consumable for on-dose or in-dose authentication in a reliable manner.

## Conclusion

We have combined photoluminescent natural biopolymers and dose information into an edible and imperceptible matrix code that can be used for serialization, track-and-trace solutions, anticounterfeit measures, and on-dose (or in-dose) authentication features for individual medicines at the dosage level. The reported matrix code offers additional dimensionality of distinct emission colors from fluorescent silk proteins. Such enhanced parameter space and encoding capacity can be useful for application-specific postprocessing and information storage. While most fluorescent material-based codes primarily rely on synthetic materials and polymers for inedible applications, the constituent materials of the code reported in this study are all proteins (silk fibroin and fluorescent proteins) that can be easily denatured and degraded by gastric proteolytic enzymes in the digestive system. Owing to the unique silk protein structures, this all protein-based code is not only tolerated in liquid solutions with a high alcohol content but also exhibits biocompatibility, photostability, and thermal reliability. Another immediate application of the reported edible code would be a hospital pharmacy setting by assisting in the development and production of single-unit packages and unit-dose packages to lower the risk of dispensing errors. Moreover, the proposed on-dose edible code can allow patients to take a role of custody in combating illicit pharmaceutical products and in maintaining a sustainable healthcare system. We also envision that this edible code can potentially be used for other security and cryptographic applications that require obliteration immediately after being scanned.

## Methods

### Construction of Plasmid Vector DNA for Silkworm Transgenesis

To generate transgenic silkworms, we constructed the transition vectors p3×P3-DsRed2-pFibH-eCFP, p3×P3-DsRed2-pFibH-eGFP, and p3×P3-eGFP-pFibH-mKate2 for the *piggyBac*-derived vector using the *piggyBac* transposon method.^83,86,121^ The constructed vectors with a helper vector were injected into preblastoderm embryos of silkworms. To construct the plasmids, the marker DsRed2 cDNA (eGFP cDNA for mKate2) was amplified by polymerase chain reaction (PCR) using specific primers with *Nhe*I/*Afl*II sites from pDsRed2-C1 (*Nh*eI-DsRed2-F: 5*′*-GCTAGCATGGCCTCCTCCGAGAAC-3*′* and DsRed2-*Afl*II-R: 5*′*-CTTAAGCTACAGGAACAGGTGGTGGCG-3*′*; Clontech, Mountain View, CA, USA) and was cloned into the pGEM-T Easy Vector system (Promega Co., Madison, WI, USA), designated as pGEMT-DsRed2 (pGEMT-eGFP for mKate2). The DsRed2 gene was excised from pGEMT-DsRed2 digested with restriction enzymes of *Nhe*I/*Afl*II and was replaced with the eGFP gene from p3×P3-eGFP to form p3×P3-DsRed2 for eCFP and eGFP (p3×P3-eGFP for mKate2). A DNA fragment, which contains the promoter domain (1124 base pairs (bp)) and N-terminal region (1430 bp) including the intron (972 bp) of the fibroin heavy (H) chain gene (GenBank Accession No. AF226688, nt. 61312-63870), was amplified by PCR using the genomic DNA from *Bombyx mori* and primers (pFibHN-F: 5*′*-GGCGCGCCGTGCGTGATCAGGAAAAAT-3*′* and pFibHN-R: 5*′*-TGCACCGACTGCAGCACTA GTGCTGAA-3*′*). The resultant DNA fragment was cloned into the pGEM-T Easy Vector system, named as pGEMT-pFibH-NTR. The DNA fragment containing 180 bp of the 3*′* terminal sequence of the fibroin H-chain gene open reading frame, along with an additional 300 bp of the 3*′* region of the fibroin H-chain gene (GenBank Accession No. AF226688, nt. 79021-80009), was amplified by PCR using genomic DNA from *Bombyx mori* and primers (pFibHC-F: 5*′*-AGCGTCAGTTACG GAGCTGGCAGGGGA-3*′* and pFibHC-R: 5*′*-TATAGTATTCTTAGTTGAGAAGGCATA-3*′*), and then the resultant DNA fragment was cloned into the pGEM-T Easy Vector system, designated as pGEMT-CTR. The fragments were prepared by digesting pGEMT-pFibH-NTR with *Asc*I/*Bam*HI and pGEMT-CTR with *Sal*I/*Fse*I, respectively. These two fragments were cloned with the pBluescriptII SK(−) vector (Stratagene, CA, USA) digested with *Apa*I/*Sal*I, resulting in pFibHNC-null. The eCFP, eGFP, and mKate2 genes, purchased from the BIONEER corporation (Deajeon, Republic of Korea), were synthesized. The N- and C-terminals had the *Not*I and *Sbf*I restriction sites, respectively. A fragment of the eCFP, eGFP, or mKate2 gene without a termination codon was amplified from peGFP-1 (Clontech) using primers (eGFP-F: 5*′*-GCGGCCGCATGGTGAGCAAGGGCGAGGAG-3*′* and eGFP-R: 5*′*-GCTGAGGCTTGTACAGC TCGTCCAT-3*′*) and was cloned into the pGEM-T Easy Vector system. The resultant fragment was digested with *Not*I/*Bbvc*I and subsequently cloned into pFibHNC-null digested with *Not*I/*Bbvc*I, resulting in pFibHNC-eCFP, pFibHNC-eGFP, or pFibHNC-mKate2. Each vector of pFibHNC-eCFP, pFibHNC-eGFP, and pFibHNC-mKate2 was digested with *Asc*I/*Fse*I and was subcloned into p3×P3-DsRed2 (p3×P3-eGFP for mKate2). The resultant vector was named as p3×P3-DsRed2-pFibH-eCFP, p3×P3-DsRed2-pFibH-eGFP, and p3×P3-eGFP-pFibH-mKate2, respectively.

### Regeneration of Transgenic Fluorescent Silk

To avoid heat-induced denaturation of fluorescent proteins in silk,^90,122,123^ we carried out a regeneration process of fluorescent silk under 50 °C. First, fluorescent silk cocoons were cut into small pieces less than 2–5 mm, and then the silk pieces were completely dissolved in an aqueous mixture solution of lithium bromide (9.5 M) at 50 °C for 12 h with stirring of 400 rpm. The dissolved solution was filtered through a miracloth. To remove salt, the solution was dialyzed with a cellulose semipermeable tube in deionized water at room temperature for at least 2 days, exchanging deionized water several times. Finally, we obtained eCFP silk, eGFP silk, and mKate2 silk fibroin solutions with a final concentration of 5–6% (w v^–1^). The regenerated silk fibroin solutions were stored at 4 °C in the dark before use. For typical wild-type white silk, however, we followed the conventional dissolution process of silk fibroin reported in the previous protocol.^124^

### Fabrication of Multidimensional Fluorescent Codes

To develop a three-dimensional (3D) code of three distinct fluorescence emission colors, we formed three different matrix code patterns with predetermined openings on a thin nonfluorescent white silk fibroin film patterned with micrograting arrays. Each matrix code pattern was fabricated using eCFP silk, eGFP silk, or mKate2 silk fibroin solutions. A matrix code pattern of nonfluorescent white silk fibroin was also added to have four different types of signals. Each silk fibroin solution was sequentially coated on the planar side of the patterned silk fibroin film using a doctor blade method. Vinyl masks (thickness = 80 μm) with four different opening patterns were prepared by a Cricut Explore Air 2 cutter (Cricut, Inc., South Jordan, UT, USA). After coating, the samples were cured under ambient conditions in the dark. Each coating process was repeated two times. The average square size and thickness of fluorescent silk codes are 700 and 70 μm, respectively. After drying, the masks were carefully removed, resulting in edible codes with 5 × 5, 7 × 7, and 9 × 9 matrix arrays, depending on the final size of 6 × 6, 9 × 9, and 11 × 11 mm^2^, respectively.

### Digitized Key Extraction of an Edible Code Using Deep Neural Networks

We used a convolutional neural network (CNN) method to extract a binary output key from a raw fluorescence image (Table S1). Our facile fabrication process of edible matrix codes is advantageous on the laboratory scale but is subject to generate individual square units with slightly different shapes (Figure S7). Instead of using conventional image processing (e.g., edge detection), we utilized a 2D CNN model for extracting a binary output key to overcome any imperfections of matrix code patterns resulting from the facile fabrication process. The 2D CNN model is designed as follows: for a 7 × 7 matrix code, an input fluorescence image (692 pixels × 648 pixels) is convolved with 16 filters with a size of 32 × 32 and a stride of 2 in the first layer. The second convolutional layer consists of 32 filters with a size of 16 × 16 and a stride of 1. In the third convolutional layer, 64 filters with a size of 8 × 8 and a stride of 1 are applied. At each convolutional layer, batch normalization is employed for efficient and accurate training. We used the rectified linear unit (ReLU) as an activation function after each batch normalization. Max-pooling is also performed with a stride of 2 after each activation, applying the pooling size of 32 × 32, 16 × 16, and 8 × 8 in the first, second, and third convolutional layers, respectively. After the flattening step, two fully connected layers are constructed. For the first layer with 400 nodes, batch normalization and ReLU activation are applied. The second layer of 49 nodes returns an output key of 49 bits for a 7 × 7 matrix code. The 2D CNN model was learned with the mean squared error over a maximum of 15 epochs. We used the ADAM optimization to train the networks with an initial learning rate of 2 × 10^–4^ and a mini-batch size of 100. For modeling and learning, we established customized codes using MATLAB (R2021a, MathWorks, Natick, MA, USA) with Deep Learning Toolbox on NVIDIA GeForce RTX 3090 GPU (Santa Clara, CA, USA).

### Data Augmentation for a Training Data Set and Validation of the 2D CNN Model

Training of the 2D CNN model required an extremely large number of different matrix code patterns. On the other hand, actual production of a large quantity was limited on the laboratory scale. We augmented the training data set by synthetically forming different matrix codes from a variety of individual square units. First, we fabricated 200 individual square units following the same fabrication process, acquired fluorescence images under the custom-built imaging system, and cropped individual square units (101 pixels ×101 pixels) separately (Figure S7). These individual square units were randomly selected and were placed in a 7 × 7 matrix to generate 9494 different synthetic matrix code patterns (692 pixels × 648 pixels) that serve as the training data set to the 2D CNN model. To validate the designed 2D CNN model, we additionally generated 50 000 synthetic fluorescence images in a 7 × 7 matrix format (Figure S8). The binary output key extraction was performed in the same manner as the training process. Finally, to quantitatively evaluate the performance of the 2D CNN model, we calculated a bit error ratio from output keys extracted from 50 000 synthetic fluorescent input images. A low bit error ratio of 1.62 × 10^–4^ supports the reliability of the 2D CNN model. In other words, the 2D CNN model was successfully trained to determine individual square units and empty areas as 1’s and 0’s, respectively.

### Biodegradability of Fluorescent Silk Fibroin Films

We characterized the enzymatic denaturation and degradation of fluorescent silk proteins using eGFP silk fibroin films with a thickness of 70 μm and a size of 9 × 9 mm^2^. As gastric proteolytic enzymes, pepsin and trypsin were used. 0.1% pepsin in a phosphate buffer (pH 2.2) with 4 M urea and 3 M guanidine HCl and 0.25% trypsin in a phosphate buffer (pH 7.2) were prepared, respectively. For controls, pH 2.2 and pH 7.2 phosphate buffers were employed. All the solutions were prewarmed at 37 °C before the experiments for 10 min. Then, the eGFP silk fibroin films were immersed in each solution containing an enzyme, maintaining the temperature at 37 °C for the protein–enzyme reaction. Fluorescence images of the eGFP silk films were captured through an optical emission filter of 525 nm under a 470 nm LED light illumination with an interval time of 20 min. The enzymatic denaturation and degradation tests were repeated four times. We used repeated measures analysis of variance (ANOVA) tests to evaluate statistically significant differences. In this case, the multiple fluorescence intensity readings over time on each sample were reduced to a single response by averaging all of the readings over time to capture a representative attribute of each sample. This statistical analysis method is also known as a response feature analysis or a two-stage analysis.

### Hemolysis Test of Silk Fibroin Solutions

We conducted a red blood cell hemolysis test of white silk and fluorescent eCFP silk, eGFP silk, and mKate2 silk solutions using sheep erythrocytes. A 300 μL solution of sheep red blood cells (sheep red blood cells packed 100%, Innovative Research, Inc., Novi, MI, USA) was added to a 1 mL PBS (pH 7.2) solution and was centrifuged at 12 000 rpm for 10 min. Then, the isolated red blood cells were diluted in a 2 mL PBS solution. As a negative control, a 150 μL diluted solution of red blood cells was added to an 850 μL PBS solution (total amount of 1000 μL). A positive control was prepared by adding a 150 μL diluted solution of red blood cells to an 850 μL solution of 0.1% Triton X-100. For each silk fibroin solution of eCFP silk, eGFP silk, mKate2 silk, and white silk, a 150 μL solution with a concentration of 5–6% (w v^–1^) was added to a mixture solution of 150 μL red blood cells and 700 μL PBS. After incubation at 37 °C for 30 min, the mixture solutions were centrifuged at 12 000 rpm for 10 min. Finally, the hemolysis efficiency was calculated with the optical absorption values at λ = 580 nm for the silk samples, the positive control, and the negative control, respectively, following the standard protocol.^119^ The hemolysis test was performed under the ambient conditions in the dark: 23 ± 2 °C and 30–40% relative humidity.

### Safety Statement

No unexpected or unusually high safety hazards were encountered.

## Supporting Information Available

Experimental details including materials, chemicals, methods, and characterization; supplemental figures including transgenic silkworms and silk glands, fabrication procedure of edible matrix codes, optical properties of micrograting patterned silk fibroin films, edible matrix codes with different matrix sizes, photostability of fluorescent silk fibroin films under alcohol treatments, square units for constructing synthetic edible matrix codes, augmented fluorescence images of edible matrix codes (7 × 7) and binary output key extraction, cryptographic key generation of edible 5 × 5 and 9 × 9 matrix codes, possible quaternary and double binary keys, smartphone reader with optical filters, and stability of silk fibroin films immersed in high-value alcoholic spirits, and photostability, thermal stability, and long-term reliability of edible matrix codes; supplemental table including the 2D CNN model information for key extraction (PDF)
Movie S1: Simulated on-dose authentication of a solid oral-dosage form (e.g., pill, tablet, or capsule) using a smartphone (AVI)
Movie S2: Simulated in-dose authentication of a high-value alcoholic spirit using a smartphone (AVI)

## Supplementary Material

oc1c01233_si_001.pdf

oc1c01233_si_002.avi

oc1c01233_si_003.avi

## References

[ref1] Counterfeit Medicines; World Health Organization (WHO), Revised 14 November 2006.

[ref2] Falsified medicines: Overview. European Medicines Agency (EMA), ema.europa.eu/en/human-regulatory/overview/public-health-threats/falsified-medicines-overview (Accessed August 2021).

[ref3] Zaman M. H. Bitter Pills: The Global War on Counterfeit Drugs; Oxford University Press: New York, NY, USA, 2018.

[ref4] Mwai P. Fake Drugs: How Bad Is Africa’s Counterfeit MediButton Bush cine Problem? bbc.com/news/world-africa-51122898 (Accessed August 2020).

[ref5] Williams L.; McKnight E. The real impact of counterfeit medications. U.S. Pharm. 2014, 39, 44–46.

[ref6] Venhuis B. J.; Oostlander A. E.; Di Giorgio D.; Mosimann R.; du Plessis I. Oncology drugs in the crosshairs of pharmaceutical crime. Lancet Oncol. 2018, 19, E209–E217. 10.1016/S1470-2045(18)30101-3. 29611529

[ref7] Renschler J. P.; Walters K. M.; Newton P. N.; Laxminarayan R. Estimated under-five deaths associated with poor-quality antimalarials in sub-saharan africa. Am. J. Trop. Med. Hyg. 2015, 92, 119–126. 10.4269/ajtmh.14-0725. 25897068 PMC4455082

[ref8] Blackstone E. A.; Fuhr J. P.; Pociask S. The health and economic effects of counterfeit drugs. Am. Health Drug. Benef. 2014, 7, 216–223. PMC4105729 25126373

[ref9] Fake Drugs Kill More Than 250,000 Children a Year, Doctors Warn, The Guardian, theguardian.com/science/2019/mar/11/fake-drugs-kill-more-than-250000-children-a-year-doctors-warn, (Accessed June 2021).

[ref10] Kao S. L.; Chan C. L.; Tan B.; Lim C. C. T.; Dalan R.; Gardner D.; Pratt E.; Lee M.; Lee K. O. An unusual outbreak of hypoglycemia. New Engl. J. Med. 2009, 360, 734–736. 10.1056/NEJMc0807678. 19213693

[ref11] Griffen M. Pfizer Combats Counterfeited Viagra in Hong Kong, healthcarepackaging.com/article/pfizer-combats-counterfeited-viagra-hong-kong (Accessed July 2021).

[ref12] National Institute on Drug Abuse (NIDA), Opioid Overdose Crisis, drugabuse.gov/drug-topics/opioids/opioid-overdose-crisis (Accessed July 2021).

[ref13] Drug Enforcement Administration, DEA Issues Warning over Counterfeit Pills, dea.gov/press-releases/2021/05/21/dea-issues-warning-over-counterfeit-pills (Accessed July 2021).

[ref14] Raufu A. Influx of fake drugs to Nigeria worries health experts. Br. Med. J. 2002, 324, 698 10.1136/bmj.324.7339.698. PMC1122639 11909782

[ref15] WHO Global Surveillance and Monitoring System for Substandard and Falsified Medical Products; World Health Organization: Geneva 2017.

[ref16] Liang B. A.; Mackey T. K. Sexual medicine online risks to health-the problem of counterfeit drugs. Nat. Rev. Urol. 2012, 9, 480–482. 10.1038/nrurol.2012.148. 22824776

[ref17] Clark F. Rise in online pharmacies sees counterfeit drugs go global. Lancet 2015, 386, 1327–1328. 10.1016/S0140-6736(15)00394-3. 26460765

[ref18] Fittler A.; Vida R. G.; Radics V.; Botz L. A challenge for healthcare but just another opportunity for illegitimate online sellers: Dubious market of shortage oncology drugs. PLoS One 2018, 13, e0203185 10.1371/journal.pone.0203185. 30153304 PMC6112670

[ref19] Mackey T. K.; Nayyar G. Digital danger: A review of the global public health, patient safety and cybersecurity threats posed by illicit online pharmacies. Br. Med. Bull. 2016, 118, 110–126. 10.1093/bmb/ldw016. 27151957 PMC5127424

[ref20] Hertig J. B.; Castek S. L. The Brand Protection Professional, What Health Professionals Need to Know: Highlights from the OECD/EUIPO Report on Trade in Counterfeit Pharmaceutical Products, bpp.msu.edu/magazine/industry-sector-update-what-health-professionals-need-to-know-june2020 (Accessed July 2021).

[ref21] Hertig J. B.; Baney L.; Weber R. J. Current threats to maintaining a secure pharmaceutical supply chain in an online world. Hospital Pharmacy 2020, 55, 85–89. 10.1177/0018578719868406. 32214440 PMC7081486

[ref22] LegitScript, The Internet Pharmacy Market in 2016: Trends, Challenges, and Opportunities, safemedsonline.org/wp-content/uploads/2016/01/The-Internet-Pharmacy-Market-in-2016.pdf (Accessed June 2021).

[ref23] Subcommittee on oversight and investigations of the committee on energy and commerce house of representatives, counterfeit drugs: fighting illegal supply chains, 2nd ed., govinfo.gov/content/pkg/CHRG-113hhrg88828/pdf/CHRG-113hhrg88828.pdf, February 27 (2014) (Accessed August 2021).

[ref24] Tesfaye W.; Abrha S.; Sinnollareddy M.; Arnold B.; Brown A.; Matthew C.; Oguoma V. M.; Peterson G. M.; Thomas J. How do we combat bogus medicines in the age of the COVID-19 pandemic?. Am. J. Trop. Med. Hyg. 2020, 103, 1360–1363. 10.4269/ajtmh.20-0903. 32815510 PMC7543841

[ref25] Newton P. N.; Bond K. C.; Countries S. COVID-19 and risks to the supply and quality of tests, drugs, and vaccines. Lancet Glob. Health 2020, 8, E754–E755. 10.1016/S2214-109X(20)30136-4. 32278364 PMC7158941

[ref26] Schneider M.; Ho Tu Nam N. Africa and counterfeit pharmaceuticals in the times of COVID-19. J. Intellect. Prop. Law Pract. 2020, 15, 417–418. 10.1093/jiplp/jpaa073.

[ref27] Moyle L.; Childs A.; Coomber R.; Barratt M. J. #Drugsforsale: An exploration of the use of social media and encrypted messaging apps to supply and access drugs. Int. J. Drug Policy 2019, 63, 101–110. 10.1016/j.drugpo.2018.08.005. 30530252

[ref28] BeSafeRx: know your online pharmacy, U.S. Food and Drug Administration (FDA), fda.gov/drugs/quick-tips-buying-medicines-over-internet/besaferx-your-source-online-pharmacy-information (Accessed August, 2021).

[ref29] Alliance for Safe Online Pharmacies (ASOP) Global; buysaferx.pharmacy, www.buysaferx.pharmacy (Accessed July, 2021).

[ref30] Bansal D.; Malla S.; Gudala K.; Tiwari P. Anti-counterfeit technologies: A pharmaceutical industry perspective. Sci. Pharm. 2013, 81, 1–13. 10.3797/scipharm.1202-03. 23641326 PMC3617666

[ref31] Mackey T. K.; Nayyar G. A review of existing and emerging digital technologies to combat the global trade in fake medicines. Expert Opin. Drug Saf. 2017, 16, 587–602. 10.1080/14740338.2017.1313227. 28349715

[ref32] Bakan G.; Ayas S.; Serhatlioglu M.; Elbuken C.; Dana A. Invisible thin-film patterns with strong infrared emission as an optical security feature. Adv. Opt. Mater. 2018, 6, 1800613 10.1002/adom.201800613.

[ref33] Trenfield S. J.; Xian Tan H.; Awad A.; Buanz A.; Gaisford S.; Basit A. W.; Goyanes A. Track-and-trace: Novel anti-counterfeit measures for 3D printed personalized drug products using smart material inks. Int. J. Pharmaceut. 2019, 567, 118443 10.1016/j.ijpharm.2019.06.034. 31212052

[ref34] Ludasi K.; Jójárt-Laczkovich O.; Sovány T.; Hopp B.; Smausz T.; Andrásik A.; Gera T.; Kovács Z.; Regdon G. Jr. Anti-counterfeiting protection, personalized medicines - development of 2D identification methods using laser technology. Int. J. Pharmaceut. 2021, 605, 120793 10.1016/j.ijpharm.2021.120793. 34119582

[ref35] Drug Supply Chain Security Act Resources for State Officials, U.S. Food and Drug Administration (FDA), fda.gov/drugs/drug-supply-chain-security-act-dscsa/drug-supply-chain-security-act-resources-state-officials, Accessed: August 2021.

[ref36] MediLedger; mediledger.com (Accessed June 2021).

[ref37] Fei J.; Liu R. Drug-laden 3D biodegradable label using QR code for anti-counterfeiting of drugs. Mater. Sci. Eng. C-Mater. 2016, 63, 657–662. 10.1016/j.msec.2016.03.004. 27040262

[ref38] Edinger M.; Bar-Shalom D.; Sandler N.; Rantanen J.; Genina N. QR encoded smart oral dosage forms by inkjet printing. Int. J. Pharmaceut. 2018, 536, 138–145. 10.1016/j.ijpharm.2017.11.052. 29183858

[ref39] Felicity T. Securing each dose: Reducing falsification risk with dosage level authentication. Pharmaceut. Technol. 2021, 2021 Supplement, s29–s31.

[ref40] Altamimi M. J.; Greenwood J. C.; Wolff K.; Hogan M. E.; Lakhani A.; Martin G. P.; Royall P. G. Anti-counterfeiting DNA molecular tagging of pharmaceutical excipients: An evaluation of lactose containing tablets. Int. J. Pharmaceut. 2019, 571, 118656 10.1016/j.ijpharm.2019.118656. 31499233

[ref41] Ilko D.; Steiger C.; Keller R.; Holzgrabe U.; Meinel L. Tamper-proof tablets for distinction between counterfeit and originator drugs through PEG coding. Eur. J. Pharm. Biopharm. 2016, 99, 1–6. 10.1016/j.ejpb.2015.11.009. 26592157

[ref42] Felton L. A.; Shah P. P.; Sharp Z.; Atudorei V.; Timmins G. S. Stable isotope-labeled excipients for drug product identification and counterfeit detection. Drug Dev. Ind. Pharm. 2011, 37, 88–92. 10.3109/03639045.2010.492397. 20560792

[ref43] Grover W. H. Candycodes: Simple universally unique edible identifiers for confirming the authenticity of pharmaceuticals. medRxiv 2021, 10.1101/2021.07.30.21261395. PMC9076649 35523794

[ref44] Jeon H. J.; Leem J. W.; Ji Y.; Park S. M.; Park J.; Kim K. Y.; Kim S. W.; Kim Y. L. Cyber-physical watermarking with inkjet edible bioprinting. Adv. Funct. Mater. 2022, 2112479 10.1002/adfm.202112479.

[ref45] Fukuoka T.; Yamaguchi A.; Hara R.; Matsumoto T.; Utsumi Y.; Mori Y. Application of gold nanoparticle self-assemblies to unclonable anti-counterfeiting technology. In 2015 International Conference on Electronic Packaging and Imaps All Asia Conference (ICEP-IAAC), Piscataway, New Jersey, USA, 2015, pp 432–435.

[ref46] Smith J. D.; Reza M. A.; Smith N. L.; Gu J. X.; Ibrar M.; Crandall D. J.; Skrabalak S. E. Plasmonic anticounterfeit tags with high encoding capacity rapidly authenticated with deep machine learning. ACS Nano 2021, 15, 2901–2910. 10.1021/acsnano.0c08974. 33559464

[ref47] Smith A. F.; Skrabalak S. E. Metal nanomaterials for optical anti-counterfeit labels. J. Mater. Chem. C 2017, 5, 3207–3215. 10.1039/C7TC00080D.

[ref48] Ji X. F.; Wu R. T.; Long L. L.; Ke X. S.; Guo C. X.; Ghang Y. J.; Lynch V. M.; Huang F. H.; Sessler J. L. Encoding, reading, and transforming information using multifluorescent supramolecular polymeric hydrogels. Adv. Mater. 2018, 30, 1705480 10.1002/adma.201705480. 29356154

[ref49] Li Z. Q.; Chen H. Z.; Li B.; Xie Y. M.; Gong X. L.; Liu X.; Li H. R.; Zhao Y. L. Photoresponsive luminescent polymeric hydrogels for reversible information encryption and decryption. Adv. Sci. 2019, 6, 1901529 10.1002/advs.201901529. PMC6839628 31728289

[ref50] Liu F.; Nattestad A.; Naficy S.; Han R.; Casillas G.; Angeloski A.; Sun X.; Huang Z. Fluorescent carbon- and oxygen-doped hexagonal boron nitride powders as printing ink for anticounterfeit applications. Adv. Opt. Mater. 2019, 7, 1901380 10.1002/adom.201901380.

[ref51] Abdollahi A.; Roghani-Mamaqani H.; Razavi B.; Salami-Kalajahi M. Photoluminescent and chromic nanomaterials for anticounterfeiting technologies: Recent advances and future challenges. ACS Nano 2020, 14, 14417–14492. 10.1021/acsnano.0c07289. 33079535

[ref52] Wang H.; Ji X. F.; Page Z. A.; Sessler J. L. Fluorescent materials-based information storage. Mater. Chem. Front. 2020, 4, 1024–1039. 10.1039/C9QM00607A.

[ref53] Zhuang Y. L.; Ren X. L.; Che X. T.; Liu S. J.; Huang W.; Zhao Q. Organic photoresponsive materials for information storage: A review. Adv. Photonics 2021, 3, 014001 10.1117/1.AP.3.1.014001.

[ref54] Tan J.; Li Q. J.; Meng S.; Li Y. C.; Yang J.; Ye Y. X.; Tang Z. K.; Qu S. N.; Ren X. D. Time-dependent phosphorescence colors from carbon dots for advanced dynamic information encryption. Adv. Mater. 2021, 33, 2006781 10.1002/adma.202006781. 33709513

[ref55] Yang Y. B.; Li Q. Y.; Zhang H. W.; Liu H.; Ji X. F.; Tang B. Z. Codes in code: Aie supramolecular adhesive hydrogels store huge amounts of information. Adv. Mater. 2021, 33, 2105418 10.1002/adma.202105418. 34541727

[ref56] Li Z. Q.; Liu X.; Wang G. N.; Li B.; Chen H. Z.; Li H. R.; Zhao Y. L. Photoresponsive supramolecular coordination polyelectrolyte as smart anticounterfeiting inks. Nat. Commun. 2021, 12, 1363 10.1038/s41467-021-21677-4. 33649315 PMC7921134

[ref57] Zhang H. W.; Li Q. Y.; Yang Y. B.; Ji X. F.; Sessler J. L. Unlocking chemically encrypted information using three types of external stimuli. J. Am. Chem. Soc. 2021, 143, 18635–18642. 10.1021/jacs.1c08558. 34719924

[ref58] Zhang H. Y.; Hua D. W.; Huang C. B.; Samal S. K.; Xiong R. H.; Sauvage F.; Braeckmans K.; Remaut K.; De Smedt S. C. Materials and technologies to combat counterfeiting of pharmaceuticals: Current and future problem tackling. Adv. Mater. 2020, 32, 1905486 10.1002/adma.201905486. 32009266

[ref59] Huang C. B.; Lucas B.; Vervaet C.; Braeckmans K.; Van Calenbergh S.; Karalic I.; Vandewoestyne M.; Deforce D.; Demeester J.; De Smedt S. C. Unbreakable codes in electrospun fibers: Digitally encoded polymers to stop medicine counterfeiting. Adv. Mater. 2010, 22, 2657–2661. 10.1002/adma.201000130. 20446308

[ref60] Han S.; Bae H. J.; Kim J.; Shin S.; Choi S. E.; Lee S. H.; Kwon S.; Park W. Lithographically encoded polymer microtaggant using high-capacity and error-correctable QR code for anti-counterfeiting of drugs. Adv. Mater. 2012, 24, 5924–5929. 10.1002/adma.201201486. 22930454

[ref61] You M. L.; Lin M.; Wang S. R.; Wang X. M.; Zhang G.; Hong Y.; Dong Y. Q.; Jin G. R.; Xu F. Three-dimensional quick response code based on inkjet printing of upconversion fluorescent nanoparticles for drug anti-counterfeiting. Nanoscale 2016, 8, 10096–10104. 10.1039/C6NR01353H. 27119377

[ref62] Rehor I.; van Vreeswijk S.; Vermonden T.; Hennink W. E.; Kegel W. K.; Eral H. B. Biodegradable microparticles for simultaneous detection of counterfeit and deteriorated edible products. Small 2017, 13, 1701804 10.1002/smll.201701804. 28863234

[ref63] Liu R. R.; Jing J. B.; Zhang S.; Wang K.; Xu B.; Tian W. J.; Yang P. Aggregation-induced emission of a 2d protein supramolecular nanofilm with emergent functions. Mater. Chem. Front. 2020, 4, 1256–1267. 10.1039/D0QM00031K.

[ref64] De Jong W. H.; Borm P. J. A. Drug delivery and nanoparticles: Applications and hazards. Int. J. Nanomed. 2008, 3, 133–149. 10.2147/IJN.S596. PMC2527668 18686775

[ref65] Kumar A.; Dhawan A. Genotoxic and carcinogenic potential of engineered nanoparticles: An update. Arch. Toxicol. 2013, 87, 1883–1900. 10.1007/s00204-013-1128-z. 24068037

[ref66] Trasande L.; Liu B. Y.; Bao W. Phthalates and attributable mortality: A population-based longitudinal cohort study and cost analysis. Environ. Pollut. 2022, 292, 118021 10.1016/j.envpol.2021.118021. 34654571 PMC8616787

[ref67] Davison M. Pharmaceutical Anti-Counterfeiting: Combating the Real Danger from Fake Drugs; John Wiley & Sons, Inc.: Hoboken, NJ, USA, 2011.

[ref68] Ishiyama R.; Takahashi T.; Makino K.; Kudo Y.; Kooper M.; Abbink D., Medicine Tablet Authentication Using “Fingerprints” of Ink-Jet Printed Characters. In 2019 IEEE International Conference on Industrial Technology (ICIT); IEEE, 2019, 871–876.

[ref69] Leem J. W.; Kim M. S.; Choi S. H.; Kim S. R.; Kim S. W.; Song Y. M.; Young R. J.; Kim Y. L. Edible unclonable functions. Nat. Commun. 2020, 11, 328 10.1038/s41467-019-14066-5. 31949156 PMC6965141

[ref70] Altman G. H.; Diaz F.; Jakuba C.; Calabro T.; Horan R. L.; Chen J. S.; Lu H.; Richmond J.; Kaplan D. L. Silk-based biomaterials. Biomaterials 2003, 24, 401–416. 10.1016/S0142-9612(02)00353-8. 12423595

[ref71] Cao Y.; Wang B. C. Biodegradation of silk biomaterials. Int. J. Mol. Sci. 2009, 10, 1514–1524. 10.3390/ijms10041514. 19468322 PMC2680630

[ref72] Thurber A. E.; Omenetto F. G.; Kaplan D. L. *In vivo* bioresponses to silk proteins. Biomaterials 2015, 71, 145–157. 10.1016/j.biomaterials.2015.08.039. 26322725 PMC4573254

[ref73] Murphy A. R.; Kaplan D. L. Biomedical applications of chemically-modified silk fibroin. J. Mater. Chem. 2009, 19, 6443–6450. 10.1039/b905802h. 20161439 PMC2790051

[ref74] Liu X. F.; Zhang K. Q. Silk fiber - molecular formation mechanism, structure-property relationship and advanced applications. Oligomerization of Chemical and Biological Compounds 2014, 69–102. 10.5772/57611.

[ref75] Nguyen T. P.; Nguyen Q. V.; Nguyen V. H.; Le T. H.; Huynh V. Q. N.; Vo D. V. N.; Trinh Q. T.; Kim S. Y.; Le Q. V. Silk Fibroin-Based Biomaterials for Biomedical Applications: A Review. Polymers 2019, 11, 1933 10.3390/polym11121933. PMC6960760 31771251

[ref76] Jaramillo-Quiceno N.; Restrepo-Osorio A. Water-annealing treatment for edible silk fibroin coatings from fibrous waste. J. Appl. Polym. Sci. 2020, 137, 48505 10.1002/app.48505.

[ref77] Sun H.; Marelli B. Growing silk fibroin in advanced materials for food security. MRS Commun. 2021, 11, 31–45. 10.1557/s43579-020-00003-x.

[ref78] Ruggeri E.; Kim D. Y.; Cao Y. T.; Fare S.; De Nardo L.; Marelli B. A multilayered edible coating to extend produce shelf life. ACS Sustain. Chem. Eng. 2020, 8, 14312–14321. 10.1021/acssuschemeng.0c03365.

[ref79] Tao H.; Brenckle M. A.; Yang M. M.; Zhang J. D.; Liu M. K.; Siebert S. M.; Averitt R. D.; Mannoor M. S.; McAlpine M. C.; Rogers J. A.; Kaplan D. L.; Omenetto F. G. Silk-based conformal, adhesive, edible food sensors. Adv. Mater. 2012, 24, 1067–1072. 10.1002/adma.201103814. 22266768

[ref80] Marelli B.; Brenckle M. A.; Kaplan D. L.; Omenetto F. G. Silk fibroin as edible coating for perishable food preservation. Sci. Rep. 2016, 6, 25263 10.1038/srep25263. 27151492 PMC4858704

[ref81] Leem J. W.; Fraser M. J.; Kim Y. L. Transgenic and diet-enhanced silk production for reinforced biomaterials: A metamaterial perspective. Annu. Rev. Biomed. Eng. 2020, 22, 79–102. 10.1146/annurev-bioeng-082719-032747. 32160010

[ref82] Elick T. A.; Bauser C. A.; Fraser M. J. Excision of the *piggyBac* transposable element *in vitro* is a precise event that is enhanced by the expression of its encoded transposase. Genetica 1996, 98, 33–41. 10.1007/BF00120216. 8765680

[ref83] Tamura T.; Thibert C.; Royer C.; Kanda T.; Eappen A.; Kamba M.; Komoto N.; Thomas J.-L.; Mauchamp B.; Chavancy G.; Shirk P.; Fraser M.; Prudhomme J.-C.; Couble P. Germline transformation of the silkworm *Bombyx mori* L. using a *piggyBac* transposon-derived vector. Nat. Biotechnol. 2000, 18, 81–84. 10.1038/71978. 10625397

[ref84] Li X. H.; Burnight E. R.; Cooney A. L.; Malani N.; Brady T.; Sander J. D.; Staber J.; Wheelan S. J.; Joung J. K.; McCray P. B.; Bushman F. D.; Sinn P. L.; Craig N. L. *piggyBac* transposase tools for genome engineering. Proc. Natl. Acad. Sci. 2013, 110, E2279–E2287. 10.1073/pnas.1305987110. 23723351 PMC3690869

[ref85] Leem J. W.; Allcca A. E. L.; Kim Y. J.; Park J.; Kim S. W.; Kim S. R.; Ryu W. H.; Chen Y. P.; Kim Y. L. Photoelectric silk via genetic encoding and bioassisted plasmonics. Adv. Biosyst. 2020, 4, 2000040 10.1002/adbi.202000040. 32462817

[ref86] Leem J. W.; Choi S. H.; Kim S. R.; Kim S. W.; Choi K. H.; Kim Y. L. Scalable and continuous nanomaterial integration with transgenic fibers for enhanced photoluminescence. Mater. Horiz. 2017, 4, 281–289. 10.1039/C6MH00423G.

[ref87] Tschorn N.; Berg K.; Stitz J. Transposon vector-mediated stable gene transfer for the accelerated establishment of recombinant mammalian cell pools allowing for high-yield production of biologics. Biotechnol. Lett. 2020, 42, 1103–1112. 10.1007/s10529-020-02889-y. 32323079 PMC7275939

[ref88] Jang K. M.; Kim S. G.; Park J. Y.; Choi W. H.; Lee J. W.; Jegal H. Y.; Kweon S. J.; Choi K. H.; Park J. H. Single-dose oral toxicity study of genetically modified silkworm expressing EGFP protein in icr mouse. Korean J. Agric. Sci. 2016, 43, 109–115. 10.7744/kjoas.20160013.

[ref89] Richards H. A.; Han C. T.; Hopkins R. G.; Failla M. L.; Ward W. W.; Stewart C. N. Safety assessment of recombinant green fluorescent protein orally administered to weaned rats. J. Nutr. 2003, 133, 1909–1912. 10.1093/jn/133.6.1909. 12771338

[ref90] Kim D. W.; Lee O. J.; Kim S. W.; Ki C. S.; Chao J. R.; Yoo H.; Yoon S. I.; Lee J. E.; Park Y. R.; Kweon H.; Lee K. G.; Kaplan D. L.; Park C. H. Novel fabrication of fluorescent silk utilized in biotechnological and medical applications. Biomaterials 2015, 70, 48–56. 10.1016/j.biomaterials.2015.08.025. 26298522

[ref91] Leem J. W.; Park J.; Kim S. W.; Kim S. R.; Choi S. H.; Choi K. H.; Kim Y. L. Green light-activated photoreaction via genetic hybridization of far-red fluorescent protein and silk. Adv. Sci. 2018, 5, 1700863 10.1002/advs.201700863. PMC6010726 29938168

[ref92] Marcinkevicius P.; Bagci I. B.; Abdelazim N. M.; Woodhead C. S.; Young R. J.; Roedig U. Optically Interrogated Unique Object with Simulation Attack Prevention; Design, Automation & Test in Europe Conference & Exhibition (DATE), Florence, Italy, 2019.

[ref93] Park J.; Leem J. W.; Ku Z. Y.; Kim J. O.; Chegal W. C.; Kang S. W.; Kim Y. L. Disordered heteronanostructures of MoS_2_ and TiO_2_ for unclonable cryptographic primitives. ACS Appl. Nano Mater. 2021, 4, 2076–2085. 10.1021/acsanm.0c03367.

[ref94] Leem J. W.; Choi M.; Yu J. S. Multifunctional microstructured polymer films for boosting solar power generation of silicon-based photovoltaic modules. ACS Appl. Mater. Interfaces 2015, 7, 2349–2358. 10.1021/am5068194. 25622310

[ref95] Leem J. W.; Lee S. H.; Guan X. Y.; Yu J. S. Inverted tetrahedron-pyramidal micropatterned polymer films for boosting light output power in flip-chip light-emitting diodes. Opt. Express 2015, 23, 9612–9617. 10.1364/OE.23.009612. 25968997

[ref96] Nogueira G. M.; Rodas A. C. D.; Leite C. A. P.; Giles C.; Higa O. Z.; Polakiewicz B.; Beppu M. M. Preparation and characterization of ethanol-treated silk fibroin dense membranes for biomaterials application using waste silk fibers as raw material. Bioresour. Technol. 2010, 101, 8446–8451. 10.1016/j.biortech.2010.06.064. 20598877

[ref97] Qi Y.; Wang H.; Wei K.; Yang Y.; Zheng R. Y.; Kim I. S.; Zhang K. Q. A review of structure construction of silk fibroin biomaterials from single structures to multi-level structures. Int. J. Mol. Sci. 2017, 18, 237 10.3390/ijms18030237. PMC5372488 28273799

[ref98] Kaewpirom S.; Boonsang S. Influence of alcohol treatments on properties of silk-fibroin-based films for highly optically transparent coating applications. RSC Adv. 2020, 10, 15913–15923. 10.1039/D0RA02634D. 35493649 PMC9052366

[ref99] Parker W. A. Alcohol-containing pharmaceuticals. Am. J. Drug Alcohol. Ab. 1982, 9, 195–209. 10.3109/00952998209002622. 7171081

[ref100] Liquid Medicine May Contain a High Level of Alcohol. Use with Caution When Administering to a Child; ConsumerMedSafety (Accessed July 2021).

[ref101] Medications Containing Alcohol - A Resource Sheet, rbhmonitoring.com/Content/Oregon/Resources/Medications%20Containing%20Alcohol%20and%20Options%20Without%20Alcohol.pdf (Accessed June 2021).

[ref102] Marelli B.; Patel N.; Duggan T.; Perotto G.; Shirman E.; Li C. M.; Kaplan D. L.; Omenetto F. G. Programming function into mechanical forms by directed assembly of silk bulk materials. P. Natl. Acad. Sci. 2017, 114, 451–456. 10.1073/pnas.1612063114. PMC5255612 28028213

[ref103] Jeong L.; Lee K. Y.; Liu J. W.; Park W. H. Time-resolved structural investigation of regenerated silk fibroin nanofibers treated with solvent vapor. Int. J. Biol. Macromol. 2006, 38, 140–144. 10.1016/j.ijbiomac.2006.02.009. 16545448

[ref104] Dubey P.; Chowdhury P. K.; Ghosh S. Modulation of Aggregation of Silk Fibroin by Synergistic Effect of the Complex of Curcumin and Beta-Cyclodextrin. BBA-Proteins Proteom. 2019, 1867, 416–425. 10.1016/j.bbapap.2019.01.009. 30677520

[ref105] Preneel B. Cryptographic hash functions. Eur. T. Telecommun. 1994, 5, 431–448. 10.1002/ett.4460050406.

[ref106] Sagar F. A. Cryptographic Hashing Functions - MD5, thesis, 2016; pp 1–9. https://cs.indstate.edu/~fsagar/doc/paper.pdf

[ref107] Lachenmeier D. W.; Neufeld M.; Rehm J. The impact of unrecorded alcohol use on health: What do we know in 2020?. J. Stud. Alcohol Drugs 2021, 82, 28–41. 10.15288/jsad.2021.82.28. 33573720

[ref108] McKee M.; Adany R.; Leon D. A. Illegally produced alcohol. Br. Med. J. 2012, 344, e1146 10.1136/bmj.e1146. 22354710

[ref109] Spencer J.; Lord N.; Benson K.; Bellotti E. ‘C’ is for commercial collaboration: Enterprise and structure in the ‘middle market’ of counterfeit alcohol distribution. Crime Law Soc. Change 2018, 70, 543–560. 10.1007/s10611-018-9781-z.

[ref110] Counterfeit Goods in the Uk, Who Is Buying What, and Why; pwc.co.uk, October, 2013; p 2.

[ref111] Scotch Whisky Economic Impact Report 2018; Scotch Whisky Association, scotch-whisky.org.uk/newsroom/scotch-whisky-economic-impact-report-2018 (Accessed July 20210.

[ref112] Breg J. M.; Tymoczko J. L.; Stryer L. Section 23.1 Proteins Are Degraded to Amino Acids; Biochemistry, W. H. Freeman: New York, USA, 2002.

[ref113] Hamuro Y.; Coales S. J.; Molnar K. S.; Tuske S. J.; Morrow J. A. Specificity of Immobilized Porcine Pepsin in H/D Exchange Compatible Conditions. Rapid Commun. Mass Spectrom. 2008, 22, 1041–1046. 10.1002/rcm.3467. 18327892

[ref114] Rick W. Trypsin. In Methods of Enzymatic Analysis; Bergmeyer J. B. H. U., Grabl M., Eds.; Verlag Chemie GmbH: Weinheim, Germany, 1974, Vol. 2.

[ref115] Li X. Q.; Zhang G. H.; Ngo N.; Zhao X. N.; Kain S. R.; Huang C. C. Deletions of the aequorea victoria green fluorescent protein define the minimal domain required for fluorescence. J. Biol. Chem. 1997, 272, 28545–28549. 10.1074/jbc.272.45.28545. 9353317

[ref116] Patterson G. H.; Knobel S. M.; Sharif W. D.; Kain S. R.; Piston D. W. Use of the green fluorescent protein and its mutants in quantitative fluorescence microscopy. Biophys. J. 1997, 73, 2782–2790. 10.1016/S0006-3495(97)78307-3. 9370472 PMC1181180

[ref117] Malik A.; Rudolph R.; Sohling B. Use of enhanced green fluorescent protein to determine pepsin at high sensitivity. Analyt. Biochem. 2005, 340, 252–258. 10.1016/j.ab.2005.02.022. 15840498

[ref118] Mares R. E.; Melendez-Lopez S. G.; Ramos M. A. Acid-denatured green fluorescent protein (GFP) as model substrate to study the chaperone activity of protein disulfide isomerase. Int. J. Mol. Sci. 2011, 12, 4625–4636. 10.3390/ijms12074625. 21845100 PMC3155373

[ref119] Zhang Y. Y.; Zhao Y. L.; Song B.; Liu K. M.; Gu J. M.; Yue Y. Y.; Xiong R. H.; Huang C. B. UV-fluorescence probe for detection Ni^2+^ with colorimetric/spectral dual-mode analysis method and its practical application. Bioorg. Chem. 2021, 114, 105103 10.1016/j.bioorg.2021.105103. 34174630

[ref120] 6.15 lighting. U.S. General Services Administration, gsa.gov/node/82715 (Accessed January 2022).

[ref121] Kim S. W.; Yun E. Y.; Choi K. H.; Kim S. R.; Kang S. W.; Park S. W.; Goo T. W. Utilization of the *Bombyx mori* heat shock protein 70 promoter for screening transgenic silkworms. Entomolog.l Res. 2013, 43, 282–287. 10.1111/1748-5967.12031.

[ref122] Nagy A.; Malnasi-Csizmadia A.; Somogyi B.; Lorinczy D. Thermal stability of chemically denatured green fluorescent protein (GFP) - A preliminary study. Thermochim. Acta 2004, 410, 161–163. 10.1016/S0040-6031(03)00397-6.

[ref123] Alkaabi K. M.; Yafea A.; Ashraf S. S. Effect of pH on thermal- and chemical-induced denaturation of gfp. Appl. Biochem. Biotechnol. 2005, 126, 149–156. 10.1385/ABAB:126:2:149. 16118468

[ref124] Rockwood D. N.; Preda R. C.; Yucel T.; Wang X. Q.; Lovett M. L.; Kaplan D. L. Materials fabrication from *Bombyx mori* silk fibroin. Nat. Protoc. 2011, 6, 1612–1631. 10.1038/nprot.2011.379. 21959241 PMC3808976

