# Supplementary material for: Edible Matrix Code with Photogenic Silk Proteins
Source: ACS Cent Sci. 2022 Apr 13;8(5):513–26. doi: 10.1021/acscentsci.1c01233 (PMC9136975; doi:10.1021/acscentsci.1c01233)
Supplement: Supplementary file 1 — oc1c01233_si_001.pdf [file oc1c01233_si_001.pdf]

Supporting Information

Edible Matrix Code with Photogenic Silk Proteins

Jung Woo Leem,[1,‡] Hee-Jae Jeon,[1,‡] Yuhyun Ji,[1] Sang Mok Park,[1] Yunsang Kwak,[2] Jongwoo Park,[3] Kee-Young Kim,[3] Seong-Wan Kim,[3,] and Young L. Kim[1,4,5,6,]**

[1]Weldon School of Biomedical Engineering, Purdue University, West Lafayette, IN 47907, USA
[2]Department of Mechanical System Engineering, Kumoh National Institute of Technology, 61 Daehak-ro, Gumi-si, Gyeongsangbuk-do 39177, Republic of Korea
[3]Department of Agricultural Biology, National Institute of Agricultural Sciences, Rural Development Administration, Wanju, Jeollabuk-do 55365, Republic of Korea
[4]Purdue University Center for Cancer Research, West Lafayette, IN 47907, USA
[5]Regenstrief Center for Healthcare Engineering, West Lafayette, IN 47907, USA
[6]Purdue Quantum Science and Engineering Institute, West Lafayette, IN 47907, USA

*Authors to whom correspondence should be addressed:

Email: tarupa@korea.kr (S. -W. Kim), youngkim@purdue.edu (Y. L. Kim)

‡Contributed equally to this work

Materials and chemicals. To produce transgenic eCFP silk, eGFP silk, and mKate2 silk, we used *Bombyx mori* bivoltine strain, Baekokjam (F1 hybrid between *Jam* 123 and *Jam* 124) obtained from the National Institute of Agricultural Sciences (Wanju, Republic of Korea). For wild-type white silk, we utilized *Bombyx mori* (Baekokjam, *Jam* 123 × *Jam* 124). Chemicals and materials were used as received: dialysis tube (pore size 12000 Da MWCO), lithium bromide (LiBr, ≥ 99%), miracloth (pore size 22 – 25 μm), sodium bicarbonate ($NaHCO_3$, ≥ 99.5%), hydrogen chloride (HCl, ≥ 99%), potassium chloride (KCl), phosphate buffered saline (PBS) solution (pH 7.2), urea (8 M), guanidine hydrochloride (≥ 99%), pepsin (from porcine gastric mucosa, ≥ 250 units mg^{-1} solid), and ethyl alcohol (200 proof) were purchased from Sigma-Aldrich Co. (Milwaukee, WI, USA). Trypsin (2.5%, pH 7.2, no phenol red, Gibco™) and Triton™ X-100 (BP151-500) were purchased from Thermo Fisher Scientific (Waltham, MA, USA). 80-μm thick vinyl sheets were purchased form Cricut, Inc. (South Jordan, UT, USA). Sylgard 184 Silicone Elastomer Kit was purchased from Dow Corning Co. (Midland, MI, USA). Sheep red blood cells (100% suspension, ISHRBC100P100ML) were purchased from Innovative Research, Inc. (Novi, MI, USA). Whiskys of Jim Beam Bourbon, Jack Daniel's Old No. 7, Suntory Toki, Johnnie Walker Red Label, and Death's Door White were purchased from Walmart Inc. (West Lafayette, IN, USA). Patterned sapphire substrates were purchased from Rubicon Technology, Inc. (Bensenville, IL, USA). Deionized water (Milli-Q® system) was used. All experiments were performed under the ambient conditions: 23 ± 2 °C and 30 − 40% relative humidity.

Screening and production of transgenic silkworms. We immersed eggs obtained from female moths in a HCl solution with a specific gravity of 1.0955 at 25 °C for 30 minutes, followed by rinsing with deionized water and drying. The transition vectors p3×P3-DsRed2-pFibH-eCFP, p3×P3-DsRed2-pFibH-eGFP, or p3×P3-eGFP-pFibH-mKate2 and the helper vector pHA3PIG were dissolved in an aqueous solution of 5 mM KCl and 0.5 mM PBS (pH 7.2) at a concentration of 0.2 μg μL^{-1} and mixed at a ratio of 1:1, respectively. The mixture solution of 5 − 10 nL was injected into pre-blastoderm embryos at 2 – 8 hours post-oviposition using an IM300 microinjector (Narishige Scientific Instrument Lab., Tokyo, Japan). DNA-injected embryos were allowed to develop at 25 °C in moist chambers until hatching. The hatched larvae (Generation 0; G0) were reared and permitted to mate with each other. The resulting embryos (G1) and larvae were screened under a fluorescence microscopy (Leica, Wetzlar, Germany) using a red filter for eCFP and eGFP and a green filter for mKate2. The G1 hatched larvae (i.e., silkworms) were reared in groups and were fed with fresh mulberry leaves under the condition of 24 − 27 °C and 70 − 90% relative humidity.

Silk cocoon degumming. We eliminated sericin from silk cocoons, following the commonly available protocol of minimizing the heat-induced denaturation of fluorescent proteins (i.e., eCFP, eGFP, and mKate2) in silk.[1-3] The silk cocoons were treated several times with a 0.2% $NaHCO_3$ solution at a temperature less than 50 °C under an induced low pressure 620 mmHg, subsequently washed with deionized water several times. The degummed cocoons were naturally dried in the dark under ambient conditions.

Scanning electron microscopy (SEM). Structural morphologies were characterized by a SEM system (FEI Quanta 3D FEG) at 5 kV.

Characterization of the optical properties. We characterized the optical properties (i.e., reflectance, photoluminescence, and transmittance spectra) of samples using a fiber bundle-coupled spectrometer (VS140 VIS-NIR; Horiba Jobin Yvon Inc., Edison, NJ, USA) coupled with an integrating sphere. Absorption spectra were obtained from reflectance and transmittance (i.e., 1 − reflectance − transmittance).

Patterning of micrograting arrays on non-fluorescent white silk fibroin films via soft imprint lithography. To fabricate a white silk fibroin film patterned with micrograting arrays as a thin substrate of edible matrix codes, we used a 2-inch sapphire substrate patterned with a conical micrograting structure consisting of two-dimensional (2D) periodic hexagonal arrays. The micrograting structure with an average height of 1.5 μm and an average period of 2.9 μm has strong light diffraction, while maintaining a high transmittance value over a wide wavelength range of 300 − 1100 nm.[4,5] As an imprint stamp, an elastomeric polydimethylsiloxane (PDMS) template was utilized because of its excellent formability for micropatterns. Sylgard 184, which is composed of a silicone “T-resin” crosslinked by a mixture of vinyl-terminated PDMS and trimethylsiloxyterminated poly(methylhydro-siloxane) polymers with a ratio of 10:1 (base:agent), was poured on the patterned sapphire substrate and was cured at 75 °C for 2 hours. PDMS stamps were carefully separated from the patterned substrate, creating inverse conical micrograting patterned structures. The 5% (w v^{-1}) white silk fibroin-dissolved solution was poured on the PDMS stamp, and was cured under ambient conditions. The cured silk fibroin films were cautiously peeled off from PDMS stamps, producing a silk fibroin film patterned with conical micrograting arrays. The height and diameter of gratings are 1.4 and 2.7 μm with a distance between adjacent gratings of 2.9 μm. The thickness of patterned silk fibroin films was 100 ± 5 μm.

Construction of a custom-built mobile application (app) for reading an edible code. To read an edible code using a smartphone, we built a customized mobile app designed with Android in Java (Android studio 4.0 integrated development environment version 2.1.2, Android SDK-software development kit, and Android API Level 23), using the Open Source Computer Vision (OpenCV) library. The mobile app allows device control, image capture, preview, authentication, and hyperlink (product information).

Numerical modeling of optical diffraction from a silk firoin film patterned with micrograting arrays. We simulated optical diffraction of a silk fibroin film patterned with micrograting arrays using the finite-difference time-domain (FDTD) method (FullWAVE, Rsoft Design Group, Ossining, NY, USA) (Figure S4c). The conical microgratings on the silk fibroin film were represented by a periodic geometry in the Cartesian coordinate system by a scalar-valued function of two variables $f(x, z)$ for simplicity.[4] Assuming that the incident light enters from air into the patterned silk fibroin film at normal incidence, the amplitude of y-polarized electric field (E_y) was calculated for the incident plane wave with a Gaussian beam profile, which was normalized at $\lambda = 532$ nm. The height and period of gratings were kept at 1.4 μm and 2.9 μm, respectively. The thickness of the silk fibroin film was set to be 100 μm. The refractive index of silk fibroin was assumed to be 1.56, and the extinction coefficient was not considered because it is negligible.[6]

Photostability of fluorescent silk fibroin films under alcohol treatments. We examined the effect of alcohol solvent on the photostability of fluorescent silk films using eGFP silk fibroin films with a thickness of 70 μm and a size of 9×9 mm^2 (Figure S6). 200 proof ethyl alcohol (ethanol) was used as a test solvent. The eGFP silk fibroin films were soaked in the prepared ethanol solvents with different concentrations of 10 – 99%. For comparison, deionized water (0% ethanol) was also utilized. The photostability and deformation of eGFP silk fibroin films immersed in a solvent were monitored by taking fluorescence images at a center wavelength of 525 nm under an excitation wavelength of 470 nm.

Reading of edible codes using a custom-built imaging system and a smartphone. We acquired fluorescence images of edible codes using a customized imaging system and a smartphone (model: Samsung Galaxy S21) with a custom-built mobile app. The imaging area of the imaging system is ~ 15×15 mm^2. For optical excitation sources of the imaging system, we used ultraviolet, blue, and green light-emitting diodes (LEDs) purchased from Thorlabs Inc. (Newton, NJ, USA) with a central wavelength of 415 nm (FWHM = 14 nm), 470 nm (FWHM = 25 nm), and 530 nm (FWHM = 33 nm). A charge-coupled device (CCD) camera (Princeton Instruments PIXIS 1024B, Trenton, NJ, USA) was used to image the fluorescence emission through a 25-nm bandpass filter set of excitation (λ_{ex}) and emission (λ_{em}) bands: λ_{ex} = 410 nm and λ_{em} = 460 nm; λ_{ex} = 470 nm and λ_{em} = 525 nm; and λ_{ex} = 530 nm and λ_{em} = 630 nm with acquisition times of 30, 1, and 20 seconds for eCFP silk, eGFP silk, and mKate2 silk, respectively. The optical power was kept at 3, 5, and 8 μW mm^{-2} for 415-nm, 470-nm, and 530-nm LEDs at the surface of the samples, respectively. To read edible codes using a custom-built app, a smartphone was equipped with excitation (λ_{ex} = 470 nm) and emission (λ_{em} = 525 nm) optical filters (Figure S11).

Photostability of fluorescent silk fibroin films under simulated daylight illumination. We tested the photostability of fluorescent silk fibroin films composing edible codes using a CIE Standard Illuminant LED light source with a color temperature of 6500 K (also known as D65) that mimics daylight illumination. Fluorescent silk fibroin films regenerated from eCFP silk, eGFP silk, and mKate2 silk had a thickness of 70 μm and a size of 9×9 mm^2. To conduct accelerated photobleaching over a short period of time, fluorescent silk fibroin films were irradiated by a high intensity of 5000 lx, which is 10 times stronger than the recommended light level intensity of 500 lx at a typical office workspace.[7] The optical intensity of white LEDs was measured using a commercial light meter (LX1330B-V, Dr. Meter). The fluorescence emission of each fluorescent silk fibroin film was monitored at each peak emission wavelength of 485 nm, 525 nm, and 625 nm under the corresponding excitation wavelength of 415 nm, 470 nm, and 530 nm for eCFP silk, eGFP silk, and mKate2 silk, respectively. Reduction in the fluorescence intensity due to photobleaching was quantified by the fluorescence intensity normalized by the initial value before illumination (Figure S13).

Key extraction performance of edible codes under thermal treatments. We explored the thermal stability of fluorescent silk fibroin films and edible codes. Fluorescent silk fibroin films and edible codes were placed in an oven at different temperatures of 30 − 90 °C for three hours (Figure S14). The eCFP silk, eGFP silk, and mKate2 silk fibroin films with a thickness of 70 μm and a diameter of 13 mm were tested. The fluorescence emission of each fluorescent silk fibroin film was monitored at each peak emission wavelength of λ_{em} = 485 nm, 525 nm, and 625 nm

under excitation of λ_{ex} = 415 nm, 470 nm, and 530 nm for eCFP silk, eGFP silk, and mKate2 silk, respectively. Reduction in the fluorescence intensity due to heat-induced denaturation was quantified by the fluorescence intensity normalized by the initial value at room temperature (i.e., 23 °C). For edible codes with a 7 × 7 matrix array, we calculated bit error ratios of output keys extracted from edible codes relative to the corresponding fluorescent code patterns.

Table S1. Hyperparameters of the two-dimensional convolutional neural network (2D CNN) model for key extraction of edible matrix codes.

Layer	Component	Size
Input layer	Raw fluorescence image	692 × 648 × 1
1st convolutional layer	32 × 32 convolution, 16 filters, stride 2	331 × 309 × 16
	Batch normalization	
	ReLU[a)] activation	
	32 × 32 max pooling, stride 2	150 × 139 × 16
2nd convolutional layer	16 × 16 convolution, 32 filters, stride 1	135 × 124 × 32
	Batch normalization	
	ReLU activation	
	16 × 16 max pooling, stride 2	60 × 55 × 32
3rd convolutional layer	8 × 8 convolution, 64 filters, stride 1	53 × 48 × 64
	Batch normalization	
	ReLU activation	
	8 × 8 max pooling, stride 2	23 × 21 × 64
Fully-connected layer	400 nodes	1 × 1 × 400
	ReLU activation	
Output layer	49 nodes (49-bit output key)	1 × 1 × 49

[a)]ReLU: rectified linear unit

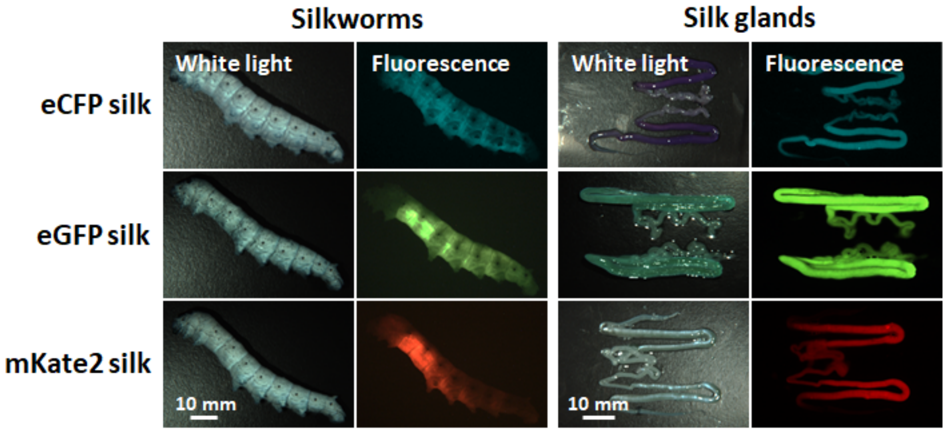

Figure S1. **Transgenic silkworms and silk glands genetically fused with enhanced cyan fluorescent protein (eCFP), enhanced green fluorescent protein (eGFP), and monomeric far-red fluorescent protein (mKate2) by the *piggyBac* transposon method**. Photographs and fluorescence images of eCFP, eGFP, and mKate2 silkworms and the corresponding silk glands. A pair of an excitation source (λ_{ex}) and an emission filter (λ_{em}) is used as follows: $\lambda_{ex}/\lambda_{em}$ = 415/460 nm, $\lambda_{ex}/\lambda_{em}$ = 470/530 nm, and $\lambda_{ex}/\lambda_{em}$ = 530/630 nm for eCFP silk, eGFP silk, and mKate2 silk, respectively.

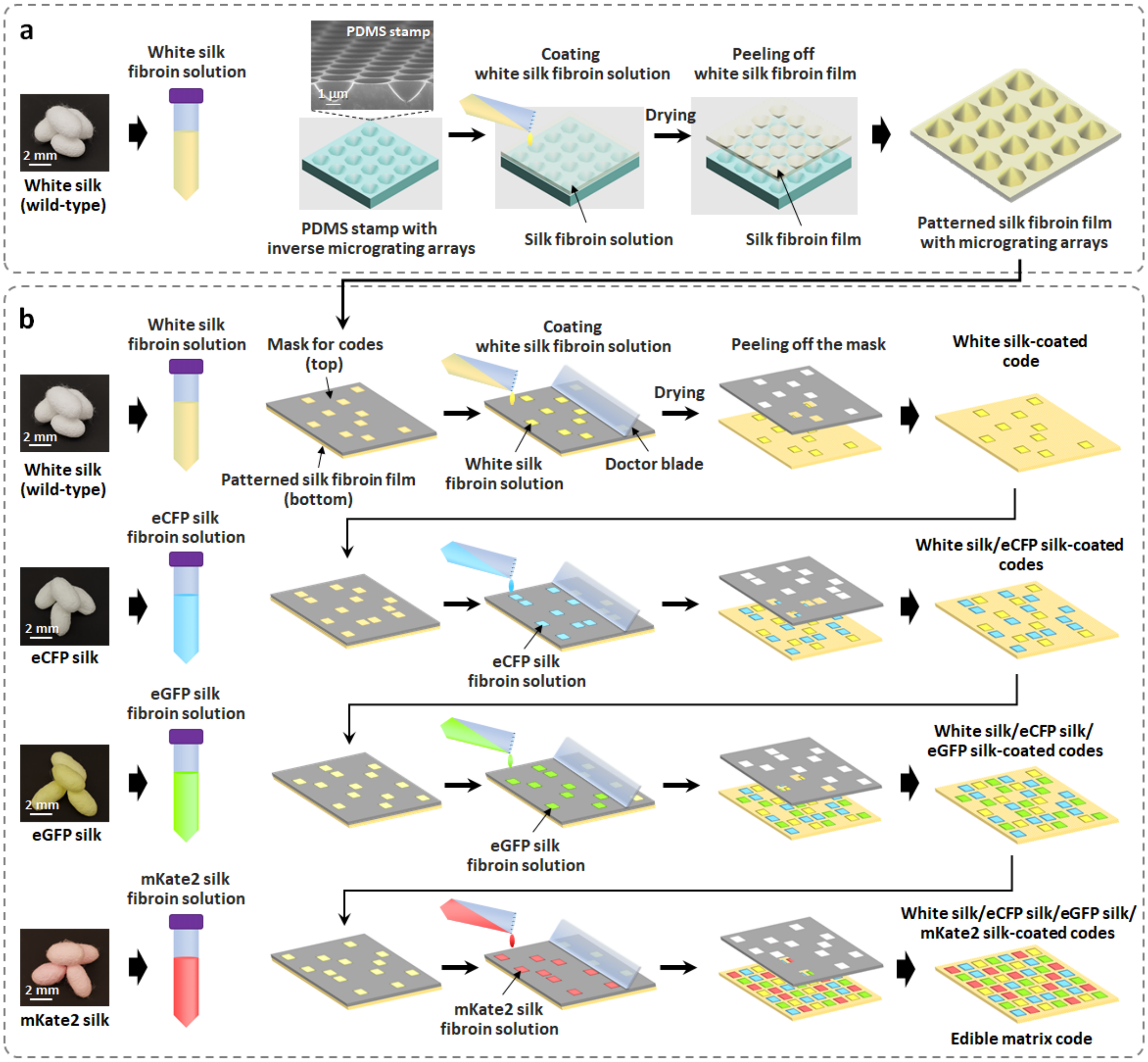

Figure S2. **Fabrication process of multidimensional codes using silk fibroin and fluorescent proteins**. (a) Schematic illustration for the fabrication process of a white silk fibroin film patterned with micrograting arrays via soft imprint lithography for the invisibility of edible codes. The haziness (optical diffraction) of the film is highly useful for the covertness of the embedded codes. Inset: SEM image of a polydimethylsiloxane (PDMS) stamp with inverted micrograting patterns. The PDMS template is used as a stamp to transfer conical micrograting arrays from a patterned sapphire substrate. (b) Schematic illustration for the fabrication process of edible codes combining three distinct fluorescence emission colors of transgenic silk and non-fluorescence emission silk. A matrix mask with pre-determined square openings is placed on a thin white silk fibroin film patterned with micrograting arrays in (a). A matrix code pattern is constructed on the planar surface side of the micrograting patterned silk fibroin film by filling in a white silk fibroin solution in the square openings with a doctor-blade method. Three additional non-overlapping matrix code patterns are constructed with eCFP silk, eGFP silk, and mKate2 silk fibroin solutions in a sequential manner.

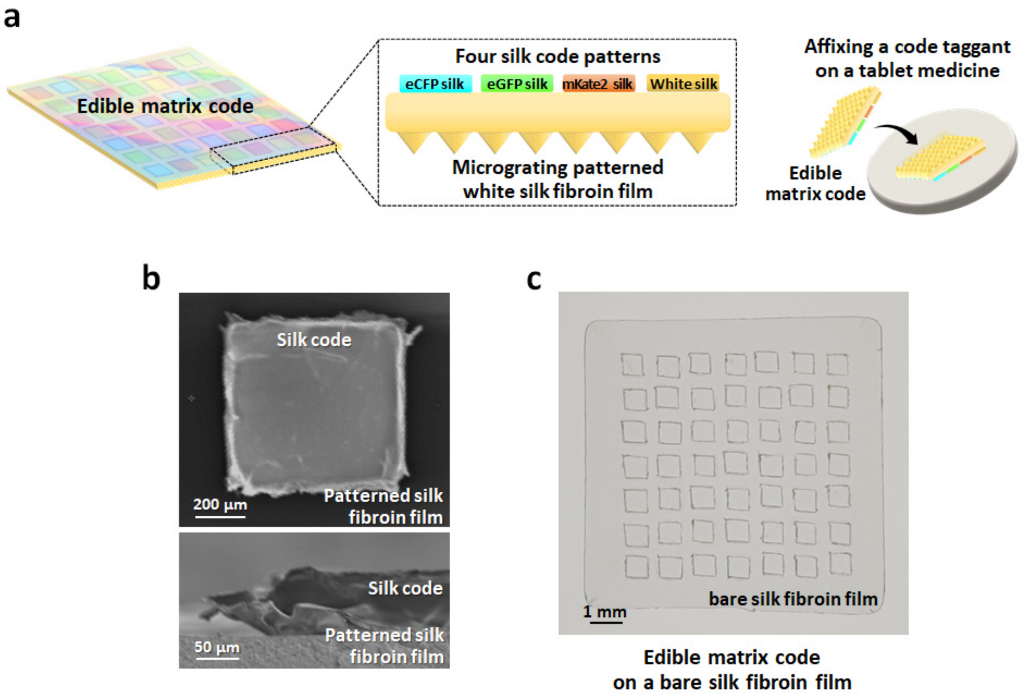

Figure S3. **Edible matrix code fabricated using silk fibroin and fluorescent proteins**. (a) Schematic illustration of an edible code formed on top of the white silk fibroin film patterned with micrograting arrays. The edible code is affixed to the medicine such that the micrograting pattern is the outermost layer. (b) Top- (upper) and side-view (lower) SEM images of a fluorescent silk code square pattern formed on the planar surface side of the silk fibroin film patterned with micrograting arrays. The thickness and size of individual fluorescent silk code patterns are 70 µm and 700 × 700 μm^2, respectively. (c) Photograph of an edible code (7 × 7 matrix array) formed on a bare silk fibroin film without micrograting patterns. In this case, the fluorescent code pattern is visible to the naked eyes.

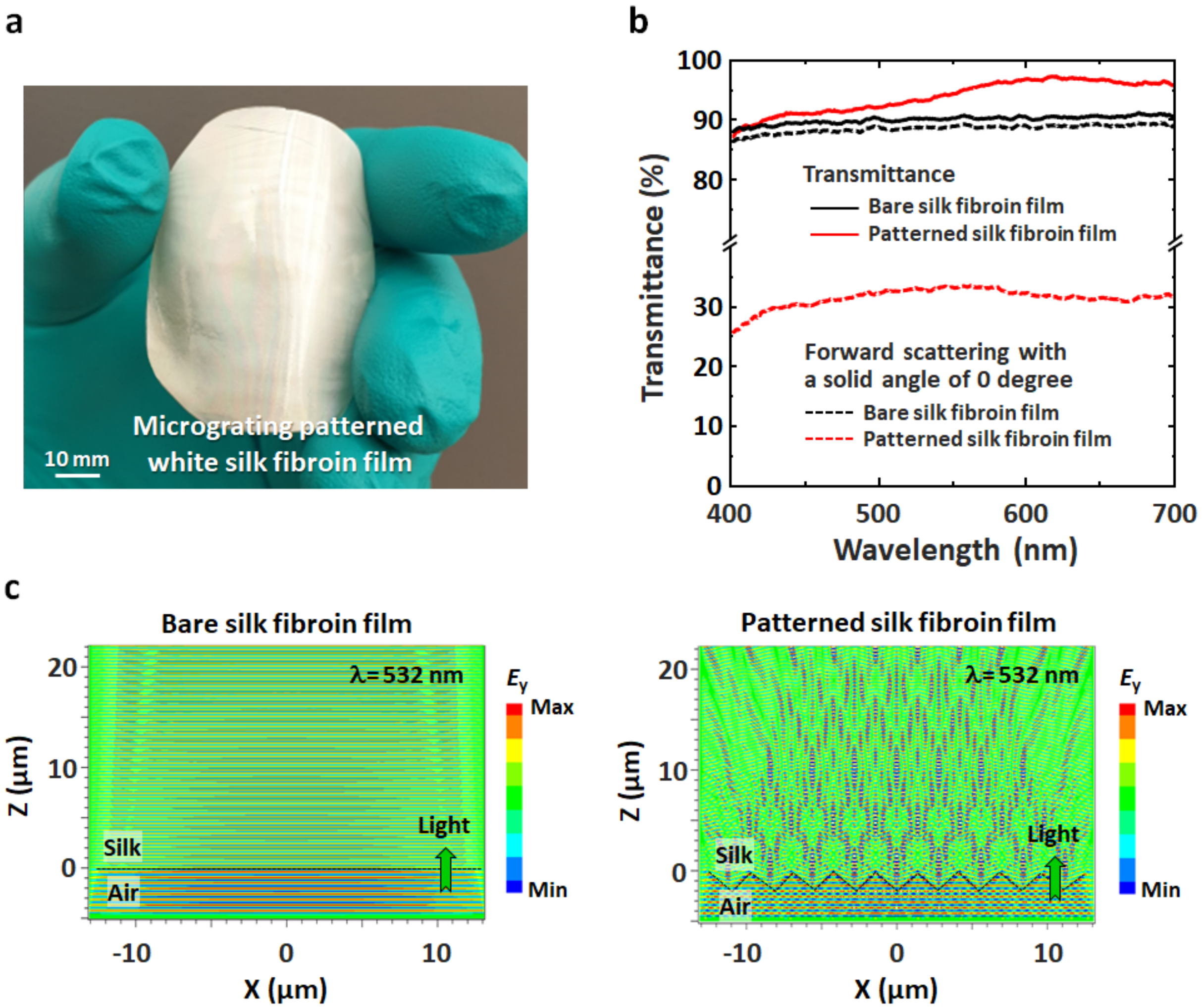

Figure S4. Optical properties of micrograting patterned white silk fibroin films. (a) Photograph of a large-area micrograting patterned white silk fibroin film with the potential for scalable production via soft imprint lithography. (b) Measured transmittance spectra of a bare silk fibroin film (black lines) and a micrograting patterned silk fibroin film (red lines). The micrograting patterned silk fibroin film does not maintain the directionality of the incident light due to optical diffraction, while the bare silk fibroin film shows no angular spread. (c) Numerical experiments of the calculated electric field (E_y) for the incident light propagating from air to a bare silk fibroin film (left) and a micrograting patterned silk fibroin film (right) under the light illumination at a wavelength (λ) of 532 nm. This simulation is conducted using the FDTD method. The electric field distribution through the conical micrograting arrays supports the experimental result that light diffraction masks the embedded code pattern, enhancing the covertness of edible matrix codes (Fig. 2h).

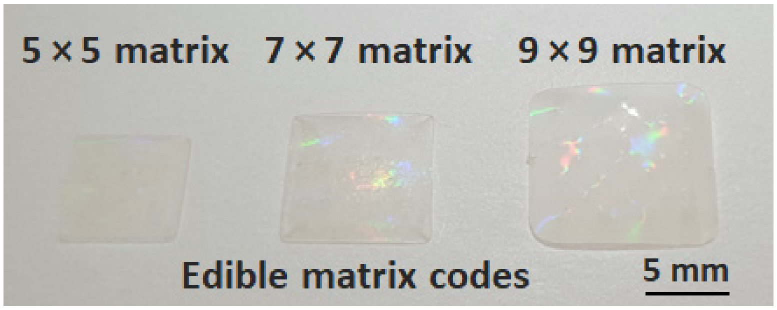

Figure S5. **Photograph of representative edible codes with 5 × 5, 7 × 7, and 9 × 9 matrix arrays**. The size of an edible matrix code can be varied by controlling the number of codes, resulting in an encoding capacity of 2^{75} (≈ 3.77×10^{22}), 2^{147} (≈ 1.78×10^{44}), and 2^{243} (≈ 1.41×10^{73}) for 5 × 5, 7 × 7, and 9 × 9 matrix codes, respectively. In these cases, the digitized key size extracted from 5 × 5, 7 × 7, and 9 × 9 matrix codes with three fluorescence colors are 75 (= 5 × 5 × 3), 147 (= 7 × 7 × 3), and 243 (9 × 9 × 3), respectively. With the individual square code pattern size of 700 × 700 μm^2, the corresponding sizes of 5 × 5, 7 × 7, and 9 × 9 matrix codes are 7 × 7, 9 × 9, and 11 × 11 mm^2, respectively.

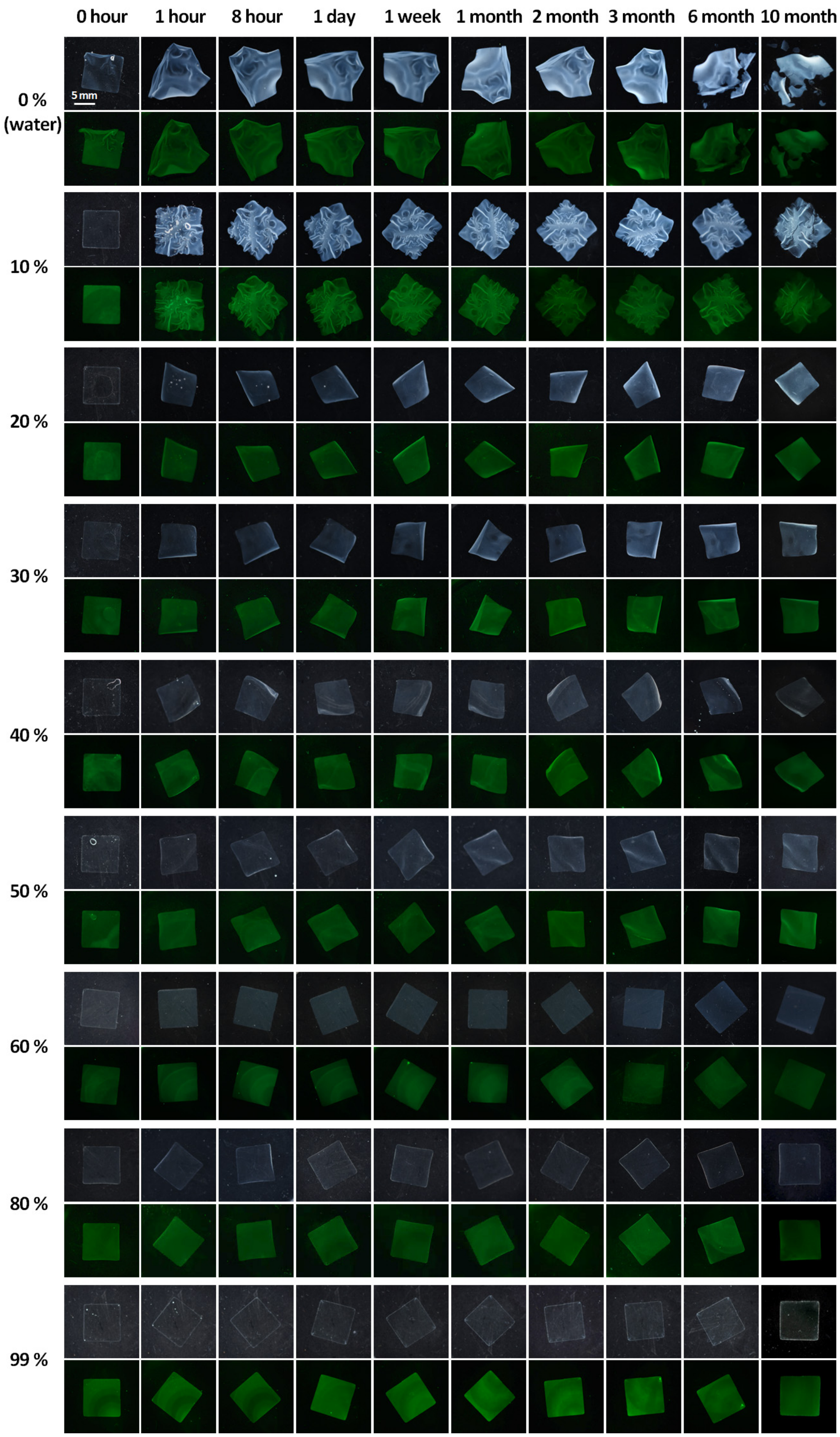

Figure S6. **Photostability of fluorescent silk fibroin films under alcohol treatments for in-dose authentication of alcohol-containing liquid type medicines**. Photographs and fluorescence images of eGFP silk fibroin films immersed in an ethanol solution at different concentrations of 0 – 99% (v v^{-1}) over an extended period of 10 months.

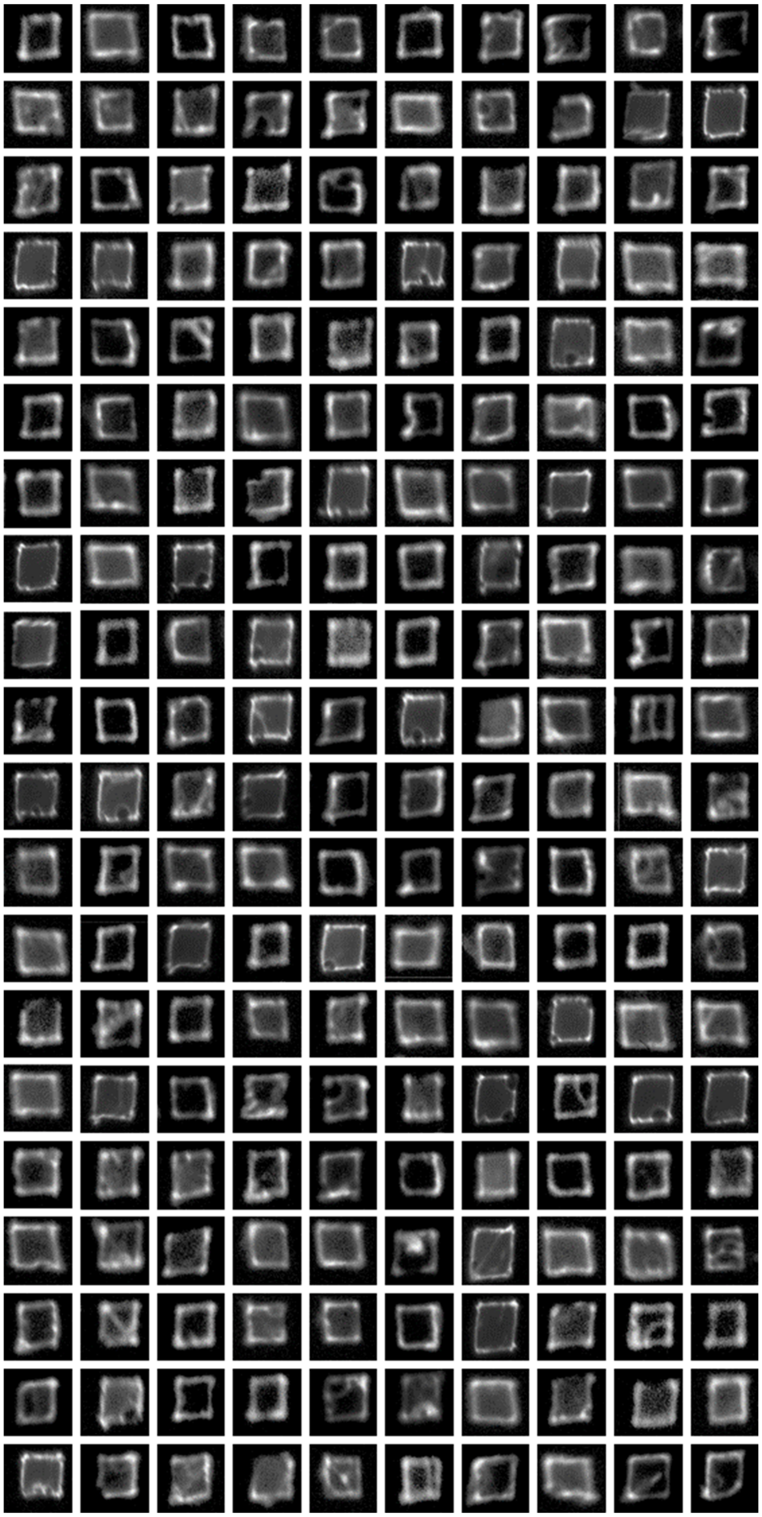

Figure S7. **Representative individual square units for constructing synthetic edible matrix codes**. 200 individual square units are fabricated following the same fabrication process. These individual square units (size ≈ 700 × 700 μm^2) are randomly selected and placed in a format of 7 × 7 matrix arrays to generate 9494 different matrix code patterns that serve as the training dataset to the 2D CNN model.

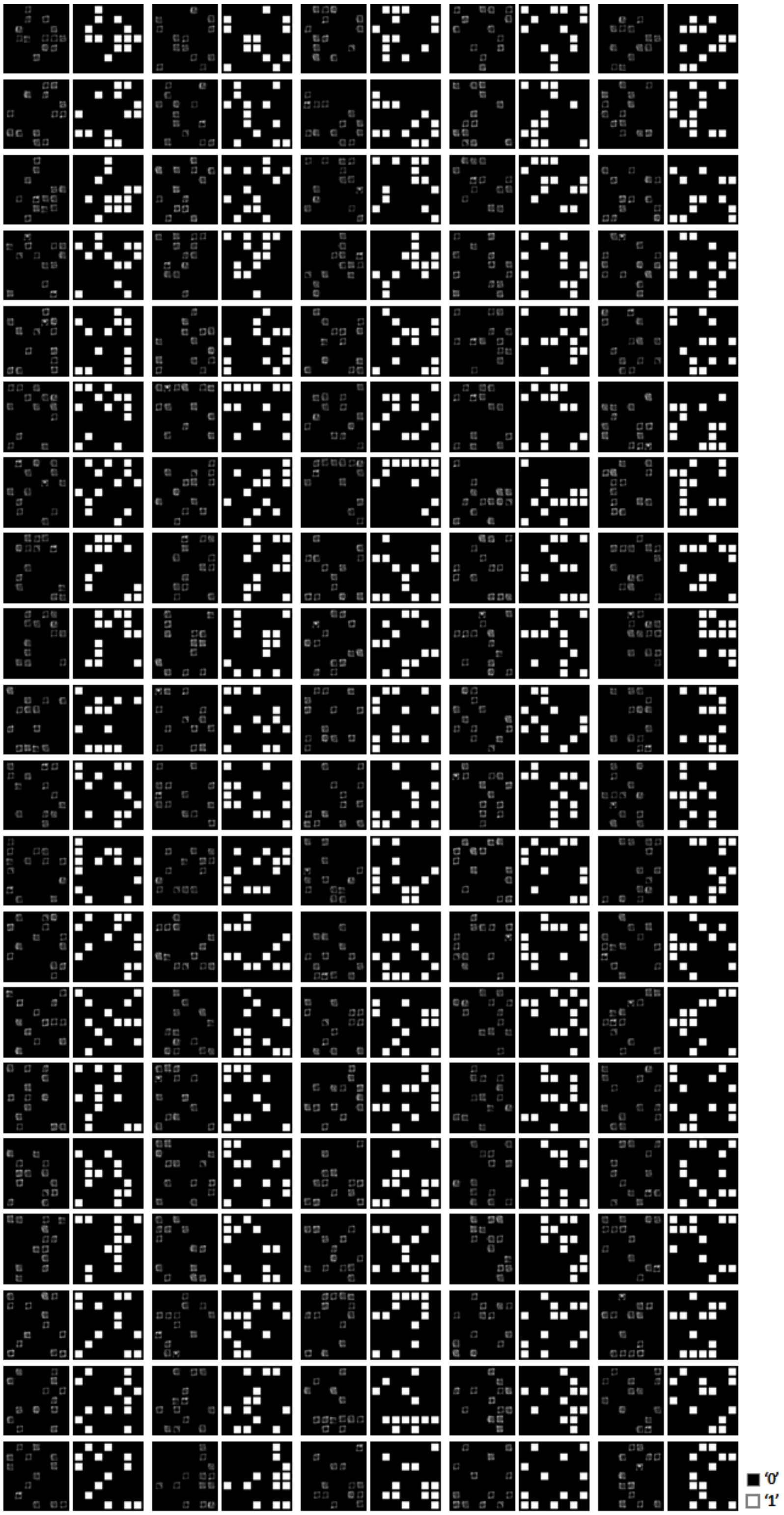

Figure S8. **Representative augmented fluorescence input images (left) of synthetic codes and binary output key (right) extraction**. For validation of the 2D CNN model, 50000 different 7 × 7 matrix codes are tested, confirming the reliable extraction of the corresponding binary output key (bitmap) that is further used to generate a security key.

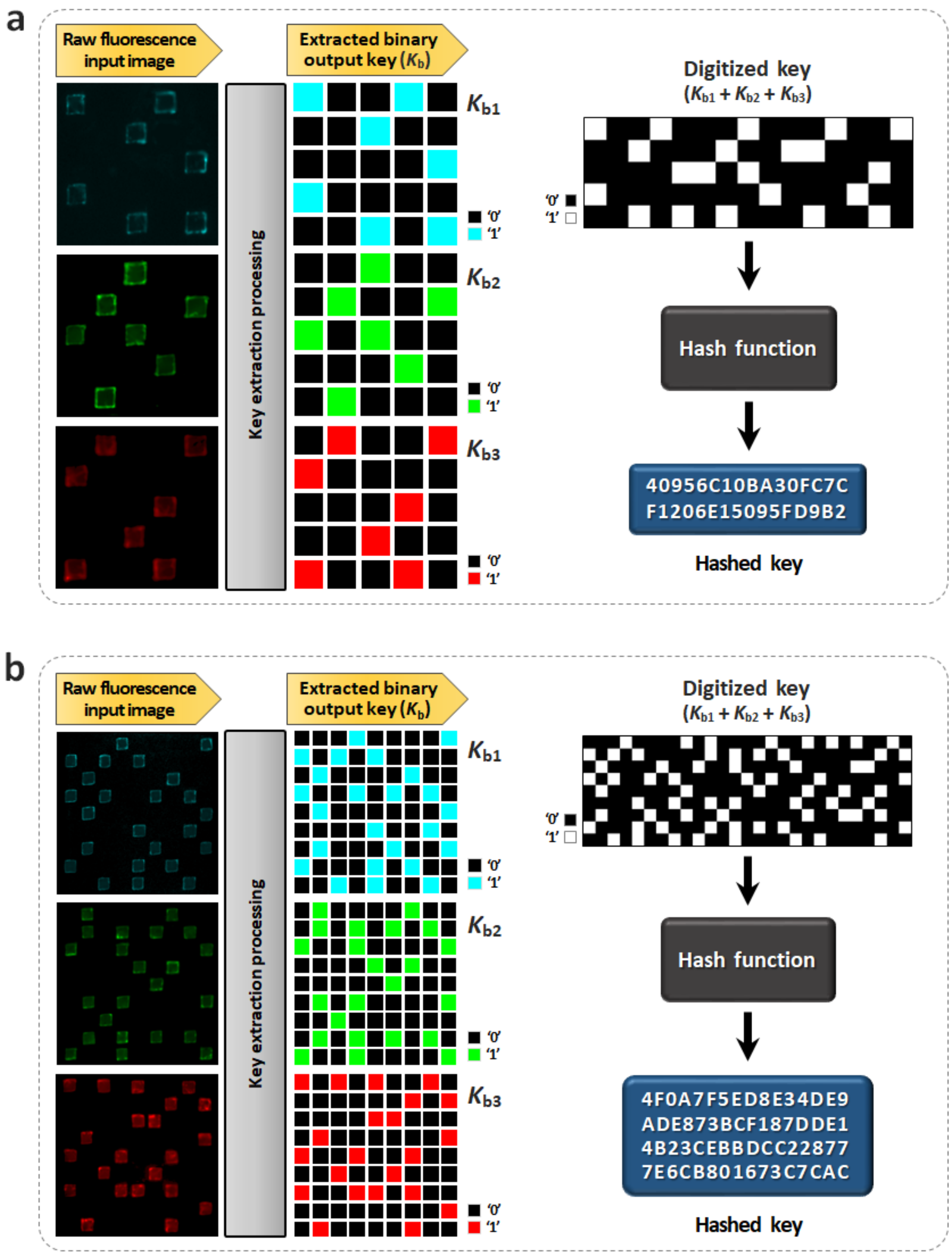

Figure S9. **Cryptographic key generation of edible 5 × 5 and 9 × 9 matrix codes with three distinct fluorescence colors and digital signature generation with a strong hash function**. Extraction process of digitized keys from fluorescence input images of a 5 × 5 matrix code (a) and a 9 × 9 matrix code (b). Three different raw fluorescence images are acquired with an optical set of an excitation source (λ_{ex}) and an emission filter (λ_{em}): eCFP silk code pattern (cyan); λ_{ex} = 415 nm and λ_{em} = 460 nm, eGFP silk code pattern (green); 470 nm and 525 nm, and mKate2 silk code pattern (red); 530 nm and 630 nm. A hashed key is further generated from the extracted digitized key via a cryptographic hash algorithm (e.g., MD5, SHA-256, or SHA-512).

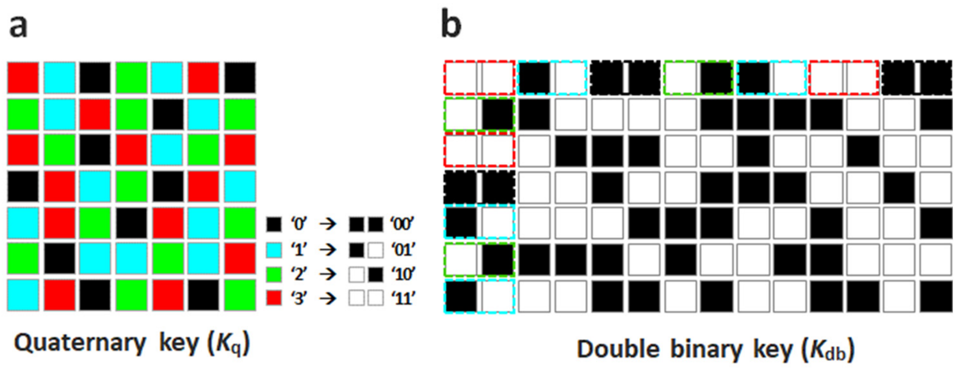

Figure S10. **Possible quaternary and double binary digitized keys reconstructed from an edible 7 × 7 matrix code with three fluorescence colors**. (a) 7 × 7 quaternary key (K_q) obtained from K_{b1}, K_{b2}, and K_{b3} in Fig. 3a, where '1' (cyan), '2' (green), and '3' (red) bits are designated from K_{b1} ('1's of eCFP silk code), K_{b2} ('1's of eGFP silk code), and K_{b3} ('1's of mKate2 silk code) and '0' (black) bits are assigned with a non-fluorescent silk code (i.e., white silk), indicating an encoding capacity of 4^{49} (≈ 3.16 × 10^{29}). (b) 14 × 7 double binary key (K_{db}) obtained from the quaternary key K_q in (a), where '00', '01', '10', and '11' bits are designated from '0', '1', '2', and '3' bits of K_q, respectively, indicating an encoding capacity of 2^{98} (≈ 3.16 × 10^{29}). This designation uses two bits to distinguish the four states from each other and results in a larger size of binary bit sequences.

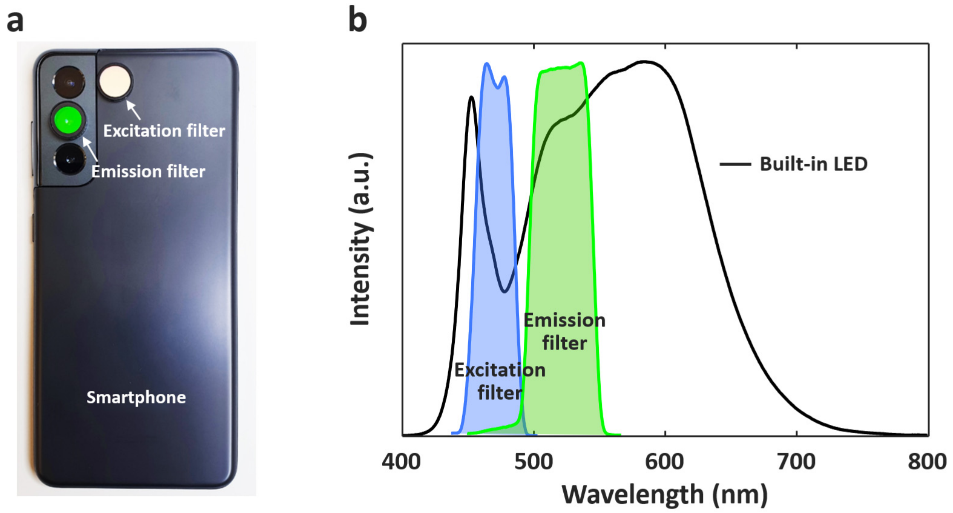

Figure S11. **Reading of edible matrix codes using a smartphone**. (a) Photograph of the smartphone used for a feasibility test. An Android smartphone (Samsung Galaxy S21) is used as a reader. (b) Spectra of the built-in flashlight LED in the smartphone and optical excitation and emission filters used for reading. This built-in LED serves as an excitation light source. Two bandpass filters for excitation (λ_{ex} = 470) and emission (λ_{em} = 525 nm) are placed in front of the flashlight LED and camera, respectively. A fluorescence image of an edible matrix code is acquired using the smartphone camera with an acquisition time of 0.2 second.

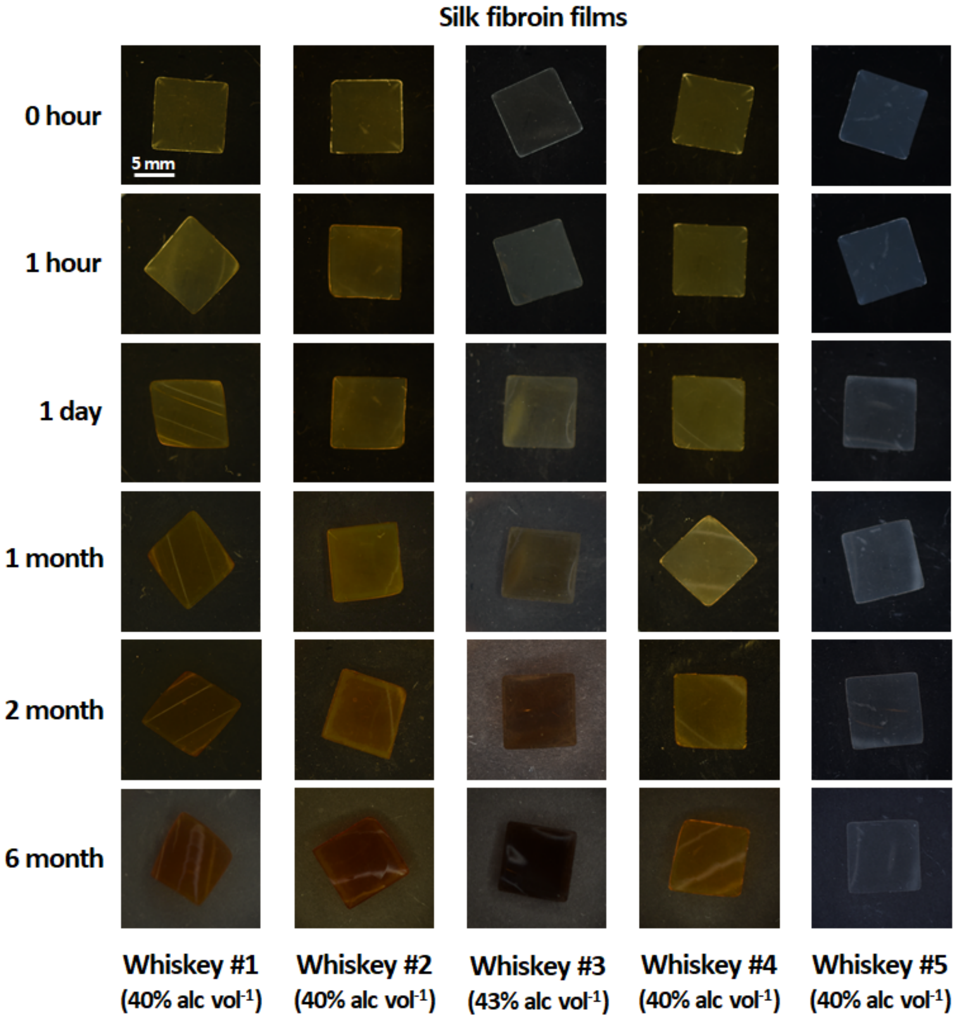

Figure S12. **Stability of silk fibroin films immersed in high-value alcoholic spirits**. Photographs of silk fibroin films immersed in five representative commercial whiskys (#1 Jim Beam Bourbon, #2 Jack Daniel's Old No. 7, #3 Suntory Toki, #4 Johnnie Walker Red Label, and #5 Death's Door White) over an extended period of six months.

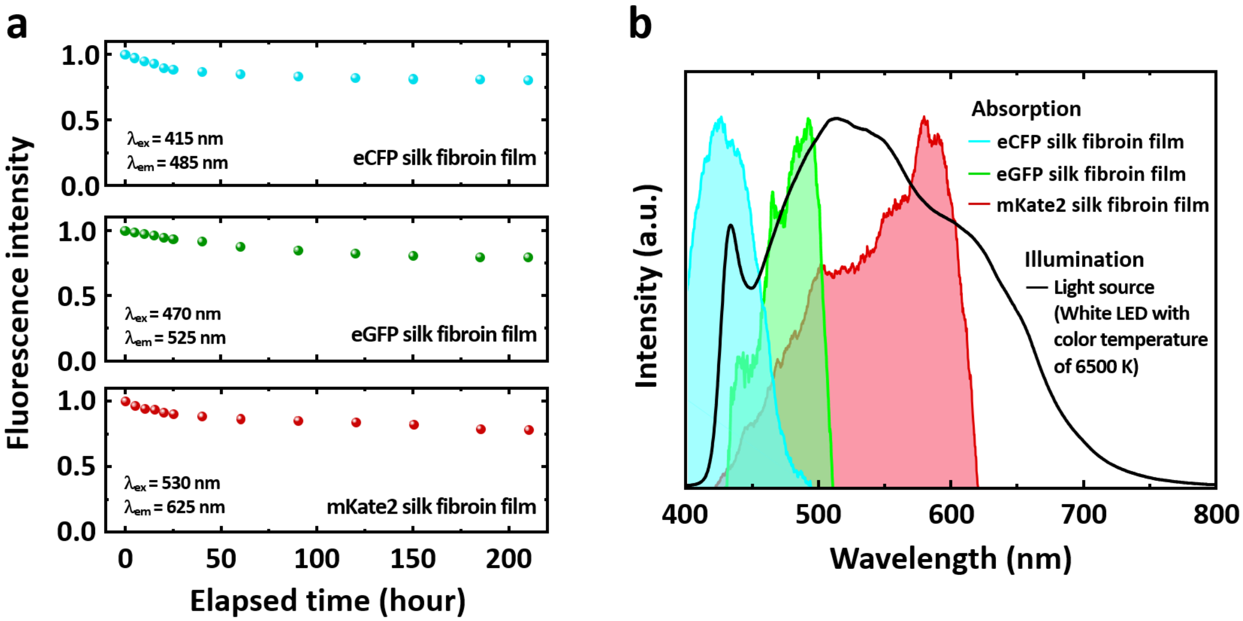

Figure S13. **Photostability of fluorescent silk fibroin films under the standard daylight illumination**. (a) Fluorescence emission intensity of fluorescent silk fibroin films as a function of elapsed illumination time. The fluorescence intensity is normalized by the value before illumination (0 hour) at each maximum emission peak (i.e., 485 nm for eCFP silk, 525 nm for eGFP silk, and 625 nm for mKate2 silk). If a currently available pharmaceutical (dark or opaque) packaging with light protection is used, the shelf life will be significantly extended. (b) Illumination spectrum of a CIE Standard Illuminant LED light source with a color temperature of 6500 K (also known as D65) used in this accelerated photobleaching experiment and absorption spectra of eCFP silk, eGFP silk, and mKate2 silk fibroin films. The D65 LED light source covers the absorption wavelength range of all fluorescent silk fibroin films and has a high illumination intensity of 5000 lx, which is 10 times higher than the recommended office workspace light intensity (i.e., 500 lx).

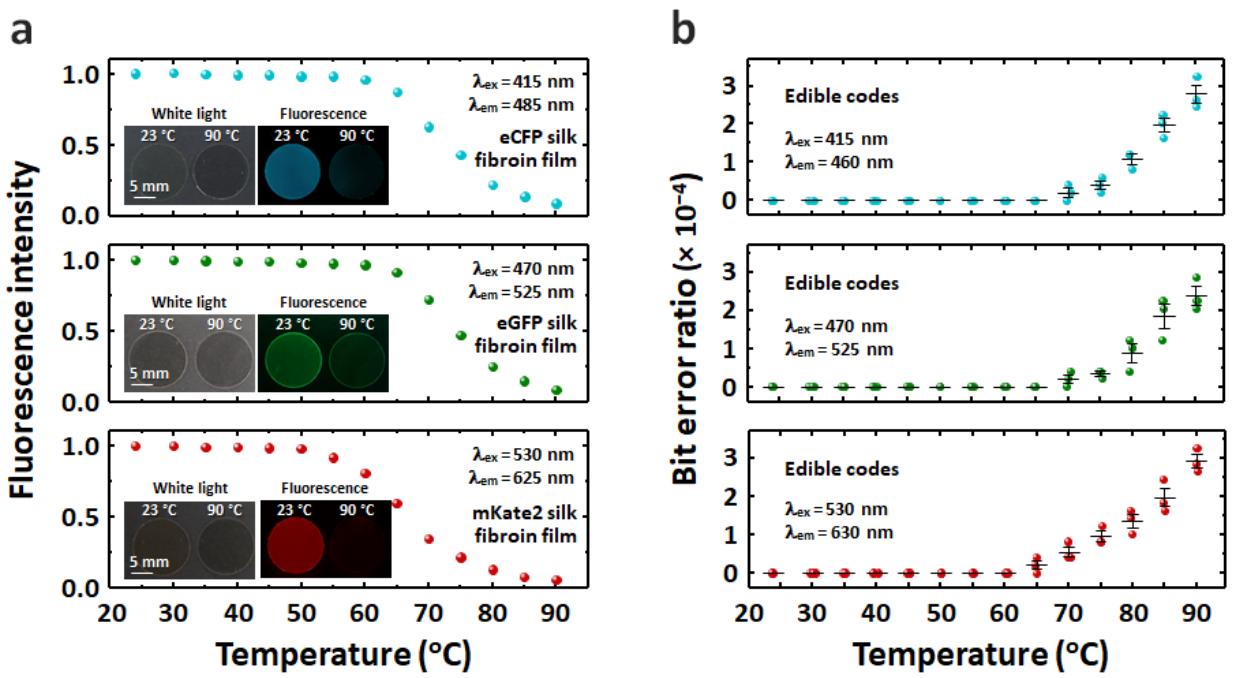

Figure S14. Photostability of fluorescent silk fibroin films and output key extraction performance of edible matrix codes under thermal treatments. (a) Fluorescence emission intensity of fluorescent silk fibroin films as a function of temperature. The fluorescence intensity is normalized by the value at a temperature of 23 °C (room temperature) for each maximum emission peak (i.e., λ_{em} = 485 nm for eCFP silk, 525 nm for eGFP silk, and 625 nm for mKate2 silk). Inset: representative photographs and fluorescence images of fluorescent silk fibroin films at temperature of 23 and 90 °C. (b) Bit error ratio of output keys extracted from edible codes at each optical set of excitation (λ_{ex}) and emission (λ_{em}) as a function of temperature. The thermal treatments are controlled by placing fluorescent silk fibroin films and edible codes in an oven at a different temperature range of 30 − 90 °C for three hours.

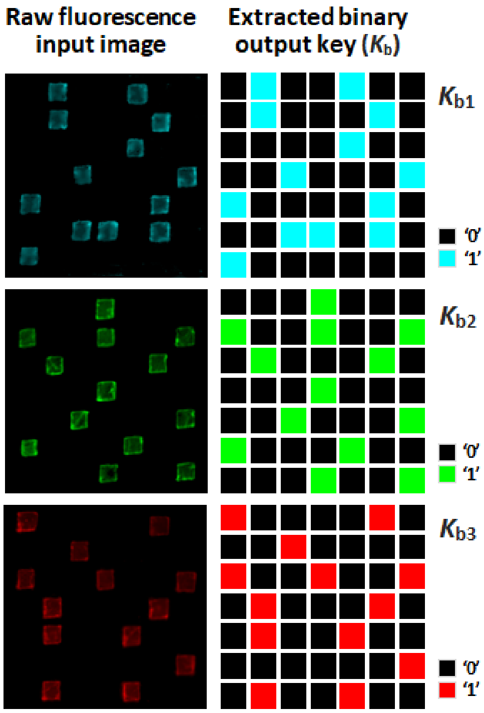

Figure S15. **Long-term reliability of edible matrix codes**. Raw fluorescent input images (left) and extracted output keys (right) acquired after 360 days, compared with those in Fig. 3a. The same optical set of excitation and emission is used: λ_{ex} = 415 nm and λ_{em} = 460 nm for eCFP silk (cyan); 470 nm and 525 nm for eGFP silk (green); and 530 nm and 630 nm for mKate2 silk (red). The storage condition is the ambient dark environment (i.e., 23 ± 2 °C and 30 – 40% relative humidity).

Supporting Information References

[1] Kim, D. W.; Lee, O. J.; Kim, S. W.; Ki, C. S.; Chao, J. R.; Yoo, H.; Yoon, S. I.; Lee, J. E.; Park, Y. R.; Kweon, H.; Lee, K. G.; Kaplan, D. L.; Park, C. H., Novel fabrication of fluorescent silk utilized in biotechnological and medical applications. *Biomaterials* **2015**, *70*, 48-56.

[2] Nagy, A.; Malnasi-Csizmadia, A.; Somogyi, B.; Lorinczy, D., Thermal stability of chemically denatured green fluorescent protein (GFP) - A preliminary study. *Thermochimica Acta* **2004**, *410*, 161-163.

[3] Alkaabi, K. M.; Yafea, A.; Ashraf, S. S., Effect of pH on thermal- and chemical-induced denaturation of GFP. *Applied Biochemistry and Biotechnology* **2005**, *126*, 149-156.

[4] Leem, J. W.; Choi, M.; Yu, J. S., Multifunctional microstructured polymer films for boosting solar power generation of silicon-based photovoltaic modules. *ACS Applied Materials and Interfaces* **2015**, *7*, 2349-2358.

[5] Leem, J. W.; Lee, S. H.; Guan, X. Y.; Yu, J. S., Inverted tetrahedron-pyramidal micropatterned polymer films for boosting light output power in flip-chip light-emitting diodes. *Optics Express* **2015**, *23*, 9612-9617.

[6] Perotto, G.; zhang, Y.; Naskar, D.; Patel, N.; Kaplan, D. L.; Cndu, S. C.; Omenetto, F. G., The optical properties of regenerated silk fibroin films obtained from different sources. *Applied Physics Letters* **2017**, *111*, 103702.

[7] U.S. General Services Administration, 6.15 lighting. gsa.gov/node/82715. Accessed: January **2022**.
